# Single-Cell RNA Sequencing Identifies Extracellular Matrix Gene Expression by Pancreatic Circulating Tumor Cells

**DOI:** 10.1016/j.celrep.2014.08.029

**Published:** 2014-09-18

**Authors:** David T. Ting, Ben S. Wittner, Matteo Ligorio, Nicole Vincent Jordan, Ajay M. Shah, David T. Miyamoto, Nicola Aceto, Francesca Bersani, Brian W. Brannigan, Kristina Xega, Jordan C. Ciciliano, Huili Zhu, Olivia C. MacKenzie, Julie Trautwein, Kshitij S. Arora, Mohammad Shahid, Haley L. Ellis, Na Qu, Nabeel Bardeesy, Miguel N. Rivera, Vikram Deshpande, Cristina R. Ferrone, Ravi Kapur, Sridhar Ramaswamy, Toshi Shioda, Mehmet Toner, Shyamala Maheswaran, Daniel A. Haber

**Affiliations:** 1 Massachusetts General Hospital Cancer Center, Harvard Medical School, Boston, MA 02114, USA; 2 Center for Engineering in Medicine, Harvard Medical School, Boston, MA 02114, USA; 3 Department of Medicine, Harvard Medical School, Boston, MA 02114, USA; 4 Department of Surgery, Harvard Medical School, Boston, MA 02114, USA; 5 Department of Radiation Oncology, Harvard Medical School, Boston, MA 02114, USA; 6 Department of Pathology, Harvard Medical School, Boston, MA 02114, USA; 7 Department of Health Sciences, University of Genoa, 16126 Genoa, Italy; 8 Howard Hughes Medical Institute, Chevy Chase, MD 20815, USA

## Abstract

Circulating tumor cells (CTCs) are shed from primary tumors into the bloodstream, mediating the hematogenous spread of cancer to distant organs. To define their composition, we compared genome-wide expression profiles of CTCs with matched primary tumors in a mouse model of pancreatic cancer, isolating individual CTCs using epitope-independent microfluidic capture, followed by single-cell RNA sequencing. CTCs clustered separately from primary tumors and tumor-derived cell lines, showing low-proliferative signatures, enrichment for the stem-cell-associated gene *Aldh1a2*, biphenotypic expression of epithelial and mesenchymal markers, and expression of *Igfbp5*, a gene transcript enriched at the epithelial-stromal interface. Mouse as well as human pancreatic CTCs exhibit a very high expression of stromal-derived extracellular matrix (ECM) proteins, including *SPARC*, whose knockdown in cancer cells suppresses cell migration and invasiveness. The aberrant expression by CTCs of stromal ECM genes points to their contribution of microenvironmental signals for the spread of cancer to distant organs.

## INTRODUCTION

Pancreatic ductal adenocarcinoma (PDAC) is a highly lethal cancer, which stems from the rapid dissemination of tumor cells leading to widespread metastasis. While local tissue and lymphatic invasion are evident even in early PDAC, the presence of circulating tumor cells (CTCs) in the bloodstream ultimately leads to spread of cancer to distant organs. CTCs are rare, estimated at one to ten tumor cells among ten billion normal blood cells in a milliliter of blood. As such, their isolation and molecular analysis has posed a significant technological challenge (Pantel et al., 2008; Yu et al., 2011). Given their role in blood-borne metastasis, CTC populations are likely to be enriched for meta-static precursors, and their analysis may identify potential therapeutic targets as well as provide opportunities for early detection of pancreatic cancer.

Genetically engineered mouse pancreatic cancer models have provided important insight into the progression of this disease. Specifically, the genetically engineered *LSL-Kras^G12D^*, *Trp53^flox/flox or +^*, *Pdx1-Cre* (KPC) mouse model recapitulates the histological progression from preneoplastic pancreatic intra-epithelial neoplasia to invasive carcinoma (Bardeesy et al., 2006). Recent studies have suggested that epithelial-to-mesenchymal transition (EMT) occurs early in this model, potentially enhancing tumor invasiveness (Rhim et al., 2012). In an initial molecular characterization of mouse pancreatic CTCs, we undertook RNA sequencing (RNA-seq) of CTC-enriched populations, identifying activation of noncanonical WNT signaling as a recurrent event, potentially contributing to the anoikis resistance of circulating epithelial cells (Yu et al., 2012). In that study, analysis of pooled CTCs, enriched from the blood but still contaminated with leukocytes, was accomplished using single-molecule RNA sequencing, combined with digital subtraction of matched leukocyte RNA reads, so as to derive a CTC-specific expression signature. However, transcriptome analysis of such partially purified cell populations is limited by depth of coverage to the most highly differentially expressed genes, and such studies of bulk CTC populations cannot resolve the degree of heterogeneity across these poorly understood cell populations.

To achieve deep RNA-sequencing profiles of CTCs at the single-cell level, we applied an inertial focusing-enhanced microfluidic device, the CTC-iChip, which allows high-efficiency negative depletion of normal blood cells, leaving CTCs in solution where they can be individually selected and analyzed as single cells (Ozkumur et al., 2013). This antigen-agnostic isolation of CTCs enables the characterization of CTCs with both epithelial and mesenchymal characteristics. Further, the high quality of RNA purified from viable, untagged CTCs is particularly well suited for detailed transcriptome analysis. We applied the CTC-iChip to the pancreatic cancer mouse model that allows for simultaneous analysis of primary tumor and CTCs, with the shared driver mutations across different animals facilitating the identification of CTC-specific heterogeneity. Here, we present a comprehensive transcriptome analysis of CTCs at the single-cell level, pointing to distinct cell subsets within CTC populations. Notably, we have identified the unexpected abundant expression of extracellular matrix (ECM) genes in mouse pancreatic CTCs and across human CTCs of pancreatic, breast, and prostate origin. Consistent with the importance of tumor stroma-derived ECM signaling in targeting cancer cell metastasis (Zhang et al., 2013), the cell-autonomous expression of ECM genes by CTCs may contribute to the dissemination of cancer to distal organs.

## RESULTS

### Isolation of Mouse Pancreatic CTCs

**Figure 1:**
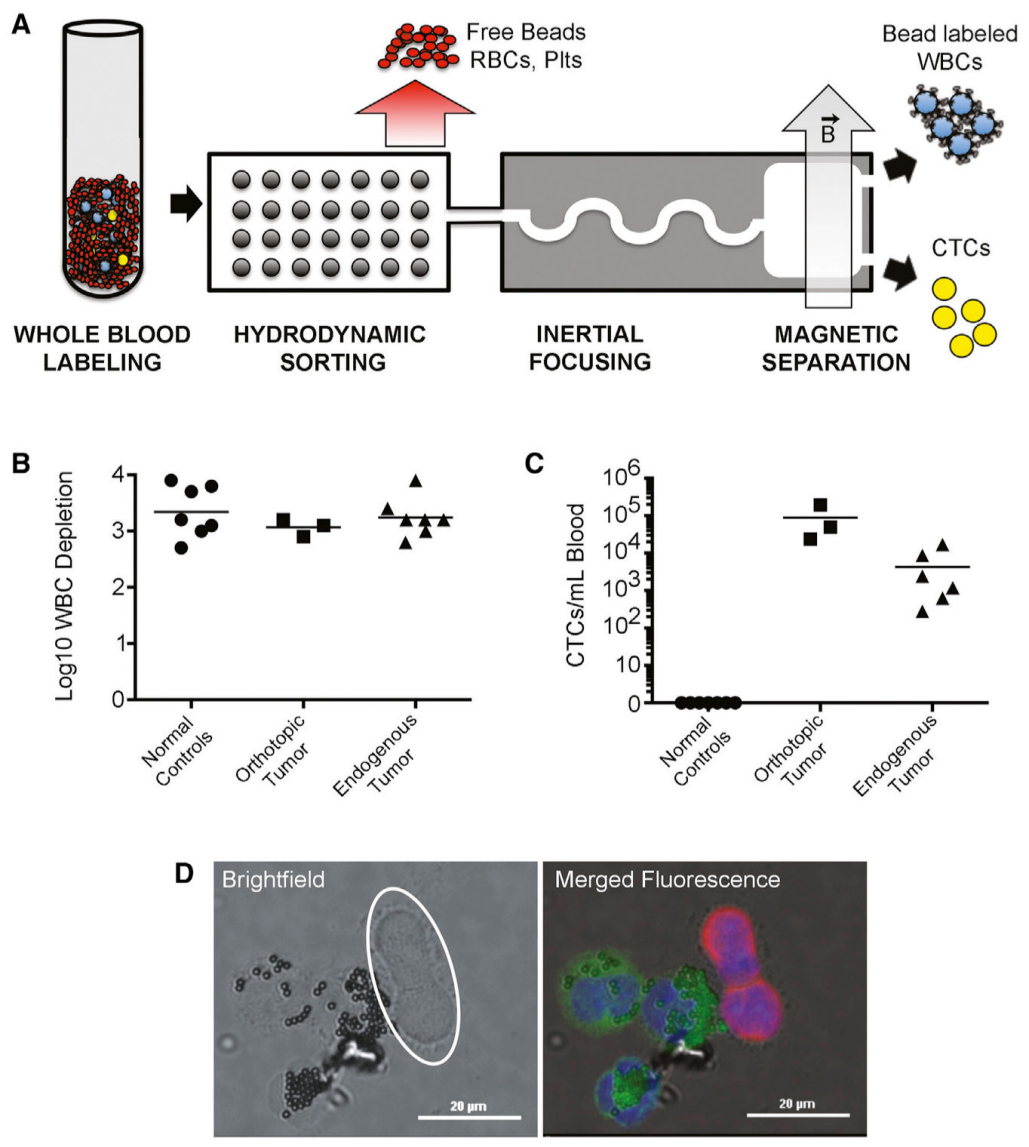
CTC Single-Cell Isolation (A) Schematic of the CTC-iChip-negative inertial focusing device system. (B) Mouse WBC depletion consistency between normal and cancer mouse models. WBC depletion is shown in log10. (C) CTC enumeration by immunofluorescent staining (CK+/CD45−/DAPI+) from normal and cancer mice. Bar represents mean. (D) Representative image of CK-positive CTCs. DAPI (blue), CK (red), and CD45 (green). Scale bar, 20 μm. Bright-field image highlighting lack of immunomagnetic anti-CD45 beads on CK+ CTCs (white circle).

The CTC-iChip combines initial hydrodynamic size-based separation of all nucleated cells (leukocytes [WBCs] and CTCs) away from red blood cells, platelets, and plasma, with subsequent inertial focusing of the nucleated cells into a single streamline to achieve high-efficiency in-line magnetic sorting. While tumor epitopes are highly variable, WBC cell-surface markers are well established; applying magnetic-conjugated anti-WBC to this very high-throughput microfluidic cell-separation device can thus exclude the vast majority of WBCs to reveal a small number of untagged CTCs (Figure 1A). Whole-blood labeling using 100 anti-CD45 beads per WBC achieved >10^3^ depletion in normal mice, mice bearing orthotopic tumors, and the KPC mice (Figure 1B).

We first tested the efficacy of the CTC-iChip using a GFP-tagged mouse PDAC cell line (NB508). CTC recovery through the CTC-iChip was measured to be 95% (mean ± 3% SD), using GFP-tagged NB508 cells spiked into whole mouse blood. Applying the CTC-iChip to orthotopic tumors derived from pancreatic inoculation of GFP-tagged NB508 cells generated >1,000 CTCs/ml in all three mice tested (Figure 1C). Finally, CTC analysis of blood specimens from KPC mice bearing endogenous tumors, using dual immunofluorescent staining of cells with the epithelial marker pan-cytokeratin (CK) and the leukocyte marker CD45, revealed a median 118 CTCs/ml (mean 429 CTCs/ml; range, 0–1,694) (Figures 1C and 1D). No CK-positive cells were detected in seven healthy control mice. The majority of CD45-positive cells that remained in the product after blood processing through the microfluidic device retained immunomagnetic beads on their surface. Thus, the untagged cells constituting CTCs were readily distinguished from WBCs in the final CTC-iChip product (Figure 1D), enabling single-cell manipulation without additional surface epitope staining.

### Single-CTC RNA-Seq

Five tumor-bearing KPC mice generated a total of 168 single CTCs (Figure S1) that were subjected to a modified single-cell amplification and library protocol (Tang et al., 2010), followed by a screen for RNA quality (*Gapdh*, *Actb*). Of these, 75 (45%) were of sufficient quality to proceed to further amplification and library construction for next-generation sequencing. It is noteworthy that a majority of candidate CTCs (55%) appeared morphologically intact but had degraded RNA. These cells likely represent tumor cells that have lost viability in the bloodstream. Given the rapid processing of blood samples from mouse models, the minimal shear condition in the microfluidic device, and the preserved RNA quality of control cells processed identically, it is unlikely that cells underwent such damage during in vitro purification. For comparison, single-cell RNA-seq was also performed on 12 WBCs from a control mouse, 12 mouse embryonic fibroblasts (MEFs), and 16 single cells from the mouse NB508 pancreatic cancer cell line. Over 90% of single cells from NB508 and MEF cultures met criteria for sequencing quality, highlighting the high frequency of CTCs with compromised RNA templates under the same conditions. To compare CTC profiles to that of matched parental tumors harvested at the time of CTC isolation, bulk RNA from each primary tumor was diluted to 1 or 10 cell equivalents (10 or 100 pg RNA) and subjected to the same amplification and RNA-seq protocol (n = 34; minimum of eight replicates from four matched tumors).

**Figure 2:**
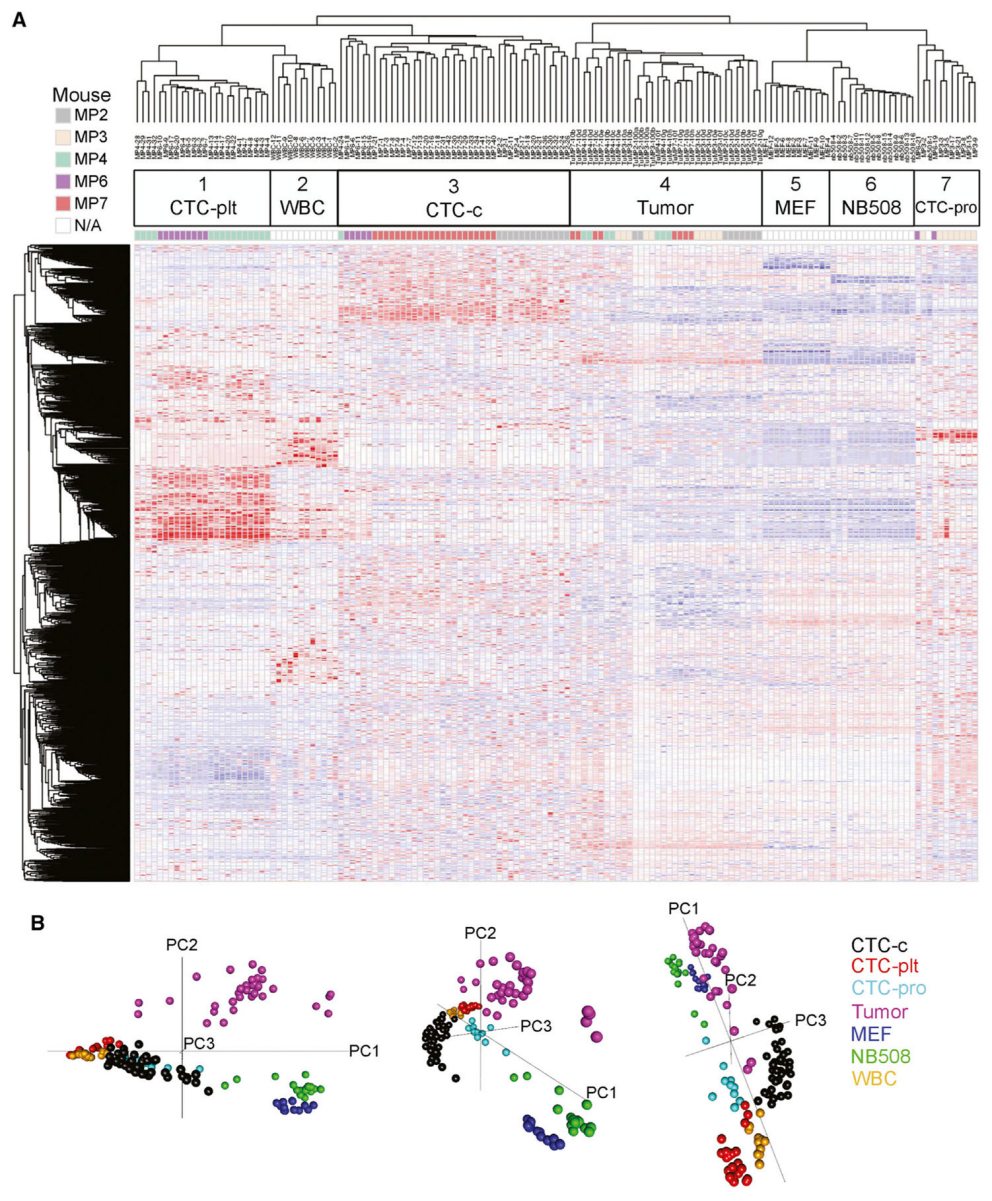
Single-Cell RNA-Seq Global Analysis (A) Unsupervised hierarchical clustering of candidate single CTCs (1, 3, and 7), single WBCs (2), single MEFs (5), single NB508 cancer cell line (6), and bulk primary tumors diluted to 10 or 100 pg of RNA (4). CTC-c, classical CTCs; CTC-plt, platelet-adhered CTCs; CTC-pro, proliferative CTCs. Data shown log transformed and median polished. (B) Principal component analysis of single-cell samples.

Single-cell RNA-seq performance was comparable for all samples analyzed, with a mean 4.4–8.5 million reads, of which a mean 46%–61% uniquely aligned to the mouse genome (Figure S1). Genome-aligned reads were annotated and counted using UCSC Known Gene transcriptome reference and normalized in reads per million (rpm). Normalized reads were then analyzed by unsupervised hierarchical clustering (Figure 2A). Single-cell transcriptomes from MEFs, the NB508 pancreatic cancer cell line, and normal WBCs clustered tightly, supporting the analytic reliability of the RNA-seq strategy. Three distinct clustering patterns of candidate CTCs were identified, all of which were distinct from matched primary tumor sequences and cancer cell lines. Principal component analysis shows the clustering and interrelationships of these different groups (Figure 2B).

The uniform genetic drivers in the KPC mouse model made it possible to quantify the degree of cellular heterogeneity in CTCs derived from individual mice and across different mice. Single-cell heterogeneity within each CTC cluster was assessed by intracluster correlation coefficients, where lower correlation coefficients reflect higher heterogeneity (Figure S1). As expected, CTC clusters showed considerably more heterogeneity (mean 0.42, 95% confidence interval [CI] 0.36–0.47) than single cells derived from the NB508 cancer cell line (mean 0.86, 95% CI 0.80–0.91, p value 1.2 × 10^−15^). To assess heterogeneity of cells within a primary PDAC, a conditional Tomato/enhanced EGFP (EGFP) (mT/mG) expression marker (Muzumdar et al., 2007) was crossed with the KPC mouse to generate a lineage-tagged mouse tumor (KPC-mT/mG) and was used to isolate individual EGFP-positive primary tumor cells away from contaminating stromal cells. A primary tumor (TuGMP3) was disaggregated into single-cell suspension, and 20 EGFP-positive cells were subjected to RNA-seq. The single primary tumor cells clustered with the previously analyzed bulk tumor material (Figure S2), with a heterogeneity score (mean 0.38, 95% CI 0.28–0.47) similar to that of CTCs (p value 0.49).

In summary, we achieved single-cell RNA-seq of mouse pancreatic CTCs isolated without positive selection bias, along with parental tumors, an established genotype-matched cancer cell line, MEFs, and WBCs. CTCs clustered separately from the primary tumor (both bulk tumor and isolated single cells) and from the tumor-derived cell line, with comparable degrees of intercellular heterogeneity between CTCs and primary tumor cells.

### Defining Subsets of Pancreatic CTCs

To identify and classify candidate CTCs, we initially applied gene sets for known epithelial, hematopoietic, and endothelial markers across all clustered samples. As expected, epithelial markers (*Krt7*, *Krt8*, *Krt18*, *Krt19*, *Epcam*, *Egfr*, *Cdh1*) were highly expressed in primary pancreatic tumors and in the cancer cell line NB508 and nearly absent in the nonepithelial MEFs and in normal WBCs (Figure S3). In contrast, hematopoietic markers (*Ptprc/Cd45*, *Csf3r/Cd114*, *Cd14*, *Fcgr3/Cd16*, *Itga2b/Cd41*, *Itgb3/Cd61*) were present in normal WBCs and absent in NB508 and MEFs. Some expression of hematopoietic markers was detectable in the bulk primary tumor samples, consistent with varying degrees of leukocytic infiltrates. No specific cluster of endothelial cells was identified, based on expression of characteristic markers (*Cdh5/Cd144*, *Vwf*, *Thbd/Cd141*, *Pecam1/Cd31*, *Mcam/Cd146*, *Sele/E-selectin*, *Cd34*) and absence of epithelial and hematopoietic markers.

**Figure 3:**
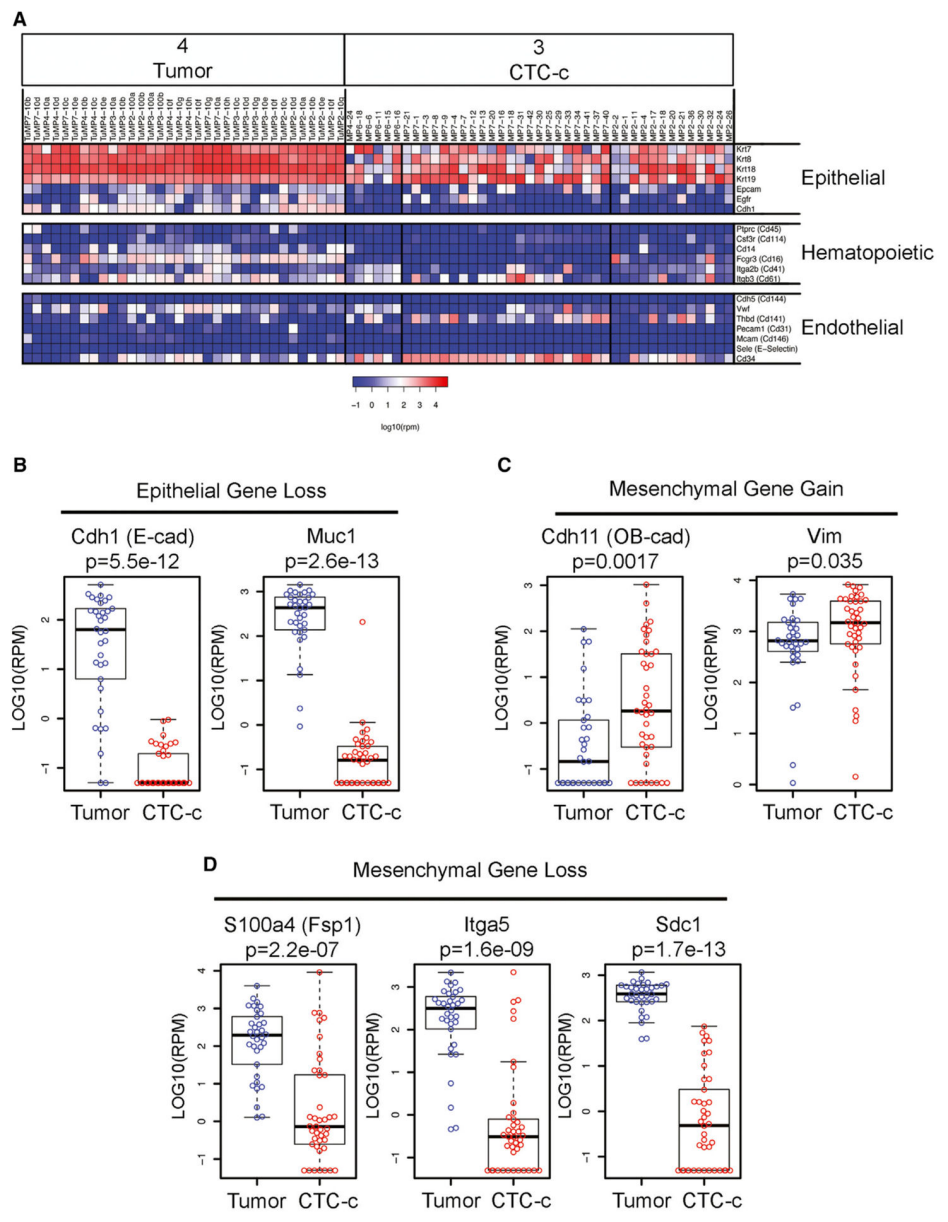
Targeted Analysis of Single-Cell RNA-Seq Data (A) Expression heatmap of epithelial, hematopoietic, and endothelial markers in primary tumors and classical epithelial CTCs (CTC-c). Scale in log10(rpm). (B–D) Epithelial and mesenchymal genes differentially expressed in CTCs versus tumors. Boxplot of epithelial genes that are (B) downregulated or mesenchymal genes that are (C) upregulated or (D) downregulated in CTCs (red) versus tumors (blue). Bar represents median, and boxplot represents quartiles; scale in log10(rpm).

Interrogation of single cells isolated by CD45 depletion from tumor-bearing mice, using the epithelial, hematopoietic, and endothelial markers, revealed notable differences among the three major candidate CTC groupings (clusters 1, 3, and 7; Figures 3A and S3). Cluster 3 showed strong expression of epithelial markers, consistent with a “classical” CTC phenotype (denoted CTC-c). A subset of these cells expressed *Cd34*, an endothelial progenitor marker that is also found in mesenchymal cells including MEFs (Figures 3A and S3) and stromal cells (Krause et al., 1994), but other characteristic endothelial lineage markers were absent. Clusters 1 and 7 were more complex, with the former noteworthy for enrichment of platelet markers CD41 (*Itga2b*) and CD61 (*Itgb3*) (hence denoted CTC-plt) and the latter having a prominent cellular proliferation signature (CTC-pro).

To better define the characteristics of each candidate CTC cluster, we used a nonparametric differential gene expression analysis including a rank product (RP) methodology adapted to variations in absolute transcript levels and differences in transcriptome representation from cell to cell (Breitling et al., 2004). Setting highly stringent parameters (false discovery rate ≤0.01), the control comparison of primary tumors versus WBCs identified 927 genes relatively overexpressed in tumors and 293 genes high in WBCs, including the expected differential expression of epithelial tumor markers keratin 7, 8, 18, and 19, versus the leukocyte-specific CD45 (Table S1). Comparing the classical CTC cluster to WBCs also showed enrichment for cytokeratin 18 and 19 in CTC-c versus CD45 in WBCs, validating the RP methodology to identify relevant differentially expressed genes between single-cell populations.

The most abundant CTC cluster, CTC-c, comprised 41 of 75 cells (55%) meeting established criteria for epithelial tumor cells (versus CTC-plt: 32%; CTC-pro: 13%). Compared with matched primary tumors, CTCs had 878 transcripts increased in expression and 774 genes with reduced expression (Table S1). Gene Ontology (GO) analysis of CTC-enriched genes (Table S2) indicated enrichment for signatures associated with cellular interactions with environmental signals (GO:0045785, positive regulation of cell adhesion), cell shape and structure (GO:0030036, actin cytoskeleton organization), and transcriptional states (GO:0045449, regulation of transcription). Kyoto Encyclopedia of Genes and Genomes (KEGG) pathway analysis (Table S3) similarly showed enrichment for focal adhesion (odds ratio [OR] 2.7, q-value 6.7 × 10^−4^) and regulation of actin cytoskeleton (OR 2.4, q-value 0.005). Notably, of the KEGG signaling pathways annotated, the mitogen-activated protein kinase (MAPK) pathway was most highly enriched (OR 2.2, q-value 0.006); MAPK signaling is already activated in the *Kras^G12D^*-driven primary tumor. However, while MSigDB Kras dependency signatures were enriched in primary tumors compared with CTCs, the latter had increased expression of *Braf*, *Mras*, and *Rras2*, pointing to alternative paths to further activate MAPK in CTCs. This finding is consistent with another study that identified the MAPK pathway as being the most highly enriched in pancreatic CTCs with microarray-based methodologies (Carvalho et al., 2013).

While single cells within the CTC cluster exhibited the characteristic features of tumor cells, defining the identity of the nonclassical CTC clusters, CTC-plt and CTC-pro, required additional analyses. Compared with CTC-c, single cells within the CTC-plt cluster were highly enriched for wound healing as well as platelet and megakaryocyte expression profiles (Table S4). While this suggests that these cells are either circulating megakaryocytes/giant platelets or CTCs covered with adherent platelets, tumor cell-specific lineage tagging supports the identification of CTC-plt cells as being of tumor origin. Eighteen EGFP lineage-tagged single CTCs from two KPC-mT/mG mice were subjected to single-cell RNA-seq: a total of nine CTCs from the two mice (seven out of seven CTCs from GMP1 and 2 out of 11 from GMP2) were included within CTC-plt using unsupervised hierarchical clustering (Figure S2). Thus, the CTC-plt cluster includes CTCs that exhibit strong platelet markers, most likely derived from transcripts encoded by adherent platelets. Interestingly, CTC-plt cells maintained their distinct segregation from CTC-c, even after digital removal of all annotated platelet transcripts (Figure S4). Thus, the adherence of abundant platelets may modulate the intrinsic CTC expression profile, as recently suggested by in vitro modeling experiments (Labelle et al., 2011).

The CTC-pro cluster was most similar to both the NB508 pancreatic cancer cell line and MEFs, and it was enriched for the cellular proliferation marker *Mki67* when compared to CTC-c. Multiple lineages are likely to have contributed to this complex grouping; nine CTCs from the two KPC-mT/mG mice described above clustered with CTC-pro (Figure S2), characterized by abundant expression of *Mki67* and an annotated cell-cycle signature (Whitfield et al., 2002) (Figure S5). One single cell within the CTC-pro cluster was derived from the pancreatic cancer cell line NB508, while another (MP3-2) had high keratin/high E-cadherin expression characteristic of classical CTCs (Figure S3). Another subcluster contained immune and dendritic cells, identified by their expression of antigen processing and presentation genes (Table S5). Taken together, the CTC-pro cluster appears to represent a grouping of highly proliferative cells, of which a subset is tumor-derived CTCs.

Together, unbiased isolation and RNA-seq evaluation of single pancreatic CTCs indicate that over half of these are nonviable with RNA at various stages of degradation. Among the remaining viable CTCs, three major classes are distinguishable by unsupervised clustering: the classical subset (CTC-c) accounts for 55%, with a second platelet-adherent group (CTC-plt; 32%) and a third heterogeneous cluster marked by proliferative signatures (CTC-pro; 13%). Given their most clearly defined tumor-derived characteristics, we selected the CTC-c cluster for detailed analysis of metastasis-associated pathways.

### Pancreatic CTCs Coexpress Epithelial, Mesenchymal, and Stem Cell Markers

The relevance of EMT to early metastasis in pancreatic cancer has been supported by lineage tracing studies in the KPC mouse (Rhim et al., 2012). We recently reported a distribution of epithelial and mesenchymal markers within individual CTCs in human breast cancer, reflecting both tumor histology and response or resistance to diverse therapies (Yu et al., 2013). To directly test for EMT in the mouse pancreatic classical CTCs, we used established epithelial (E) and mesenchymal (M) markers (Kalluri and Weinberg, 2009) to evaluate each cell within the CTC cluster (Figure S6). Compared with the primary tumor, CTC-c cells demonstrated clear loss of the epithelial markers E-cadherin (*Cdh1*) and *Muc1* (Figure 3B), whereas mesenchymal transcripts were mixed, with some showing increased expression (*Cdh11*, *Vim*) and others with reduced levels (*S100a4*, *Itga5*, *Sdc1*) (Figures 3C and 3D). Notably, even the mesenchymal genes that were up-regulated in CTCs showed a high degree of heterogeneous expression across single cells (Figure S6). In contrast, loss of E-cadherin (*Cdh1*) was nearly universal across all classical CTCs, suggesting that pancreatic CTCs indeed lose some of their epithelial characteristics.

**Figure 4:**
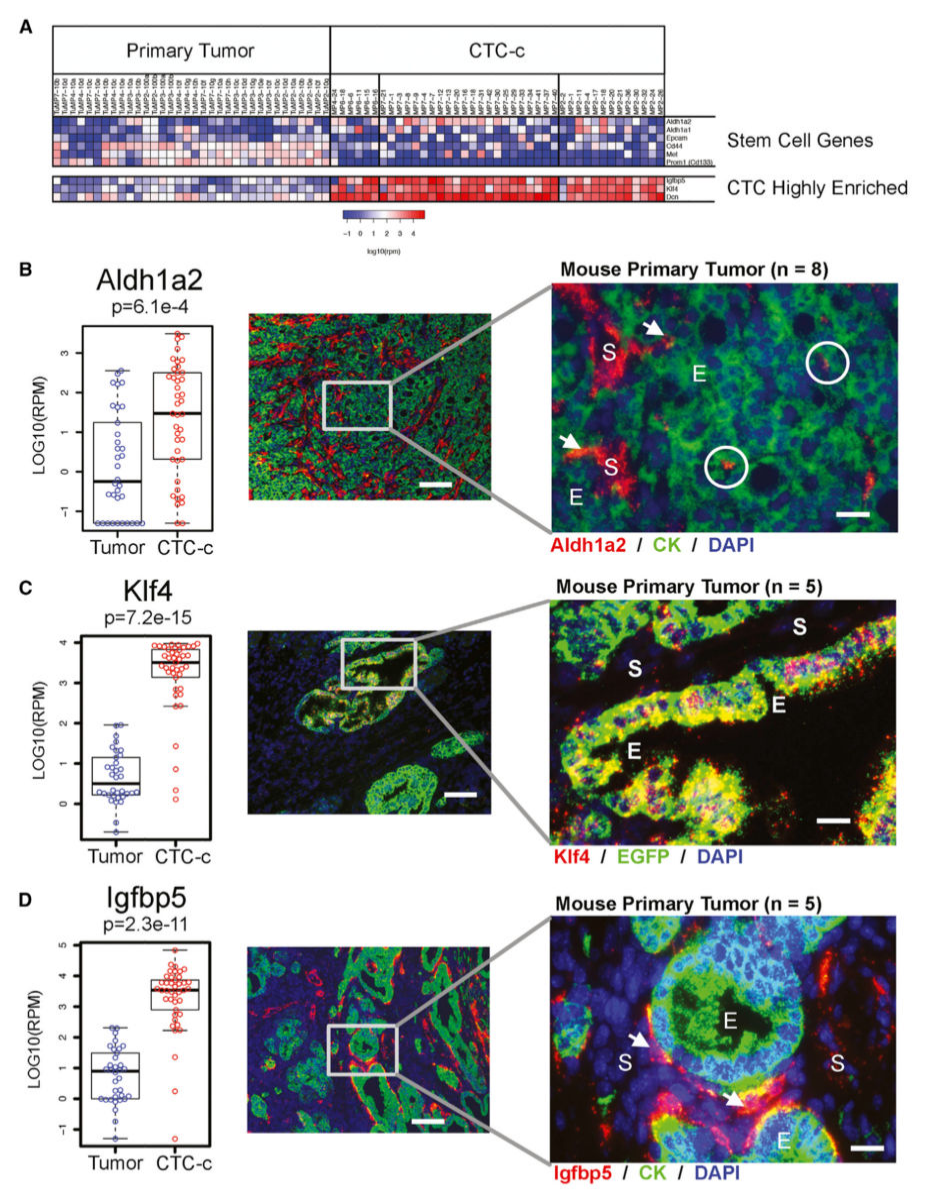
CTC-Enriched Genes Found in Epithelial and Stromal Components of Primary Tumors (A) Expression heatmap of stem cell genes and highly enriched CTC genes in primary tumors and CTC-c cells. Scale in log10(rpm). (B–D) Expression boxplot (left) analysis of (B) *Aldh1a2* stem cell and CTC highly enriched genes (C) *Klf4* and (D) *Igfbp5* genes with RNA-ISH of primary tumors (right). Bar = median, box plot = quartiles, scale in log10(rpm). RNA-ISH color key shown (CK = *Krt8+18*). Circles indicate a subpopulation of keratin-positive tumor cells with *Aldh1a2* marker, and arrowheads identify dual-positive cells at the epithelial-stromal interface (E, epithelial; S, stromal) with DAPI nuclear stain (blue). Low-magnification fluorescent images taken at 100× magnification (scale bar represents 100 μm) and high magnification at 400× (scale bar represents 20 μm).

CTCs are also likely to be enriched for metastatic precursors capable of initiating metastatic tumor deposits. The relationship between such precursor cells and cancer stem cells is uncertain, as is the relevance of established stem cell markers in identifying these cells. We evaluated putative pancreatic cancer stem cell genes (Rasheed and Matsui, 2012; Rasheed et al., 2010) in the single-cell RNA-seq reads (Figures 4A and S6). Among all candidate markers tested (*Aldh1a1*, *Aldh1a2*, *Prom1/Cd133*, *Cd44*, *Met*, *EpCAM*), only *Aldh1a1* and *Aldh1a2* were enriched in CTCs. Classical CTCs expressed predominantly the *Aldh1a2* isoform, while *Aldh1a1* was expressed in a variety of cell types (Figure S6). Within single CTCs, there was no correlation between expression of Aldh1 isoforms and either enrichment for the mesenchymal genes (*Cdh11*, *Vim*) or loss of epithelial genes (*Cdh1*, *Muc1*), suggesting that stem cell and EMT markers are not intrinsically linked in CTCs. Analysis of primary pancreatic tumors for *Aldh1a2* using RNA in situ hybridization (RNA-ISH) identified rare epithelial tumor cells expressing this stem cell marker, but the majority of expression was present within the cancer associated stromal cells (Figure 4B), consistent with immunohistochemistry for ALDH protein in human PDAC (Rasheed et al., 2010).

### Classical CTCs Share Expression of Stromal Enriched Genes

Besides the evident diversity of CTCs, we searched for shared transcripts that might provide further insight into their cell of origin within the primary tumor and the mechanisms by which they invade and survive within the bloodstream and ultimately identify potential CTC-specific therapeutic targets. We selected rigorous criteria to identify the most highly enriched CTC-c transcripts (RP score < 300), expressed at very high levels (>100 rpm) in ≥90% of all classical CTCs. Three genes met these criteria: Kruppel-like factor 4 (*Klf4*), one of the key stem cell (iPS) reprogramming factors (Takahashi and Yamanaka, 2006), which has been implicated in pancreatic cancer development (Brembeck and Rustgi, 2000; Prasad et al., 2005; Wei et al., 2010); insulin-like growth factor binding protein 5 (*Igfbp5*), an extracellular growth factor binding protein expressed in human PDAC reported to have both pro- and antiproliferative properties (Johnson et al., 2006; Johnson and Haun, 2009); and decorin (*Dcn*), a extracellular matrix proteoglycan expressed in tumor stroma across a variety of different cancers (Adany et al., 1990; Boström et al., 2013; Henke et al., 2012; Hunzelmann et al., 1995; Iozzo and Cohen, 1994; Mu et al., 2013; Nash et al., 2002). We utilized RNA-ISH in primary tumor specimens to identify the potential colocalization of these three highly enriched CTC genes. In contrast to *Aldh1a2*, *Klf4* is expressed in epithelial components of the primary tumor (Figure 4C). *Igfbp5* is of particular interest, in that it is expressed focally at the tumor epithelial-stromal interface (Figure 4D). This geographic area may be enriched for cancer cells undergoing EMT, contributing to the mixed epithelial/stromal transcriptional programs evident by RNA-seq of single CTCs.

**Figure 5:**
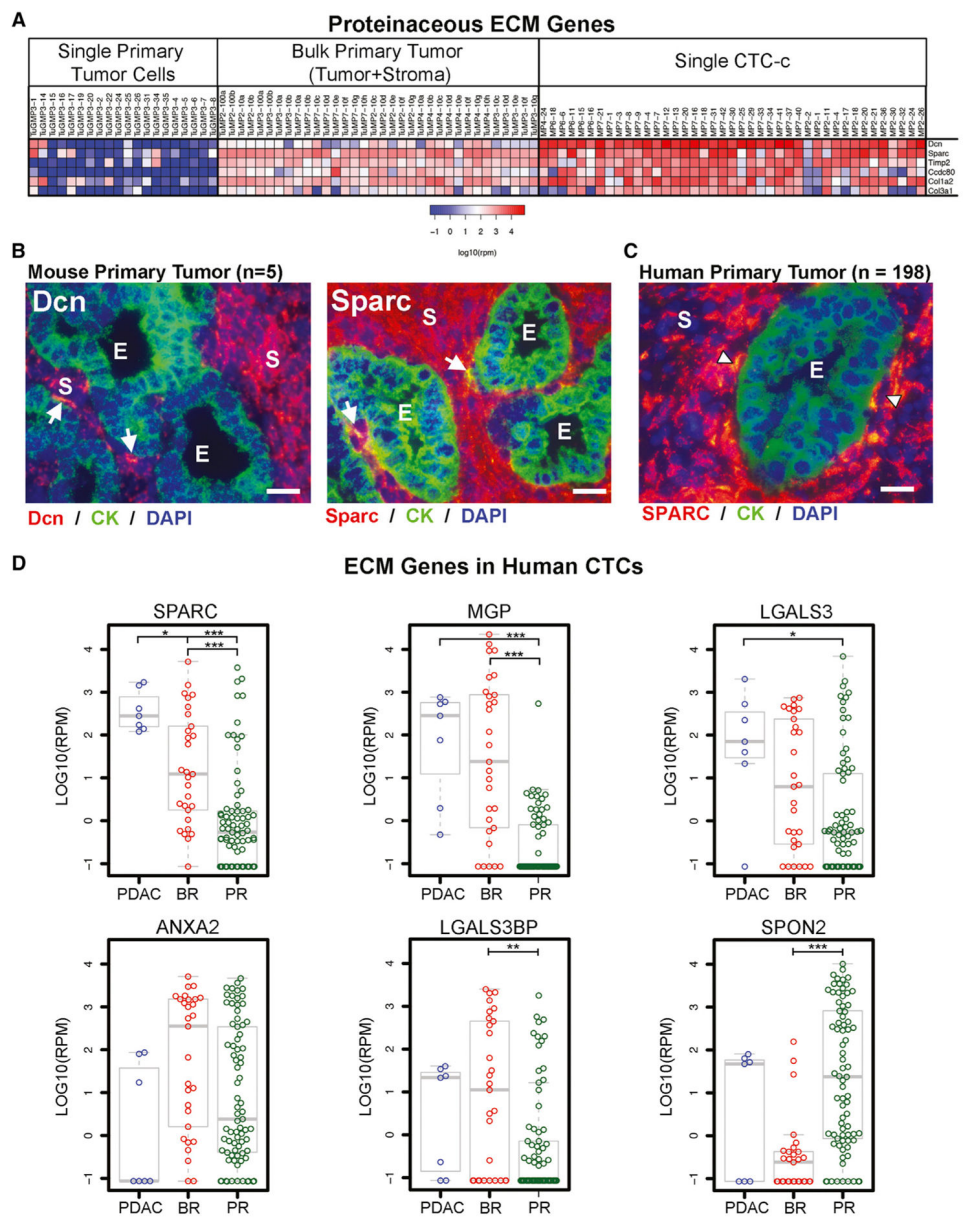
Human and Mouse CTCs across Different Epithelial Cancer Express High Levels of ECM Protein Genes (A) Expression heatmap of mouse single primary tumor cells, bulk tumor, and CTCs for ECM protein genes. Scale in log10(rpm). (B) RNA-ISH of ECM protein genes *Dcn* and *Sparc* with CK (*Krt8+18*) in mouse primary PDAC tumors. (C) RNA-ISH of *SPARC* with CK (*KRT7,8,18,+19*) in human primary PDAC tumors. Arrowheads identify dual-positive cells at the epithelial-stromal interface (E, epithelial; S, stromal) with DAPI nuclear stain (blue). Images taken at 400× magnification (scale bar represents 20 μm). (D) Expression boxplot of highly expressed ECM genes in human PDAC, breast (BR), and prostate (PR) CTCs. Bar, median; boxplot, quartiles; scale in log10(rpm). Holm-adjusted p value < 0.05 (*), 0.01 (**), 0.001 (***).

In addition to highly expressing *Dcn*, CTCs consistently had high levels of multiple ECM gene transcripts. GO analysis of all CTC-enriched genes (Table S2) identified 32 proteinaceous ECM genes (GO:0005578, OR 2.4, q-value 4.8 × 10^−3^). These genes are normally expressed in reactive stromal cells, rather than in epithelial cancer cells, and while recent studies have highlighted the importance of the stroma in supporting pancreatic cancer pathogenesis and metastasis (Feig et al., 2012; Neesse et al., 2011, 2013; Olive et al., 2009; Provenzano et al., 2012), the expression of these stroma-associated ECM genes within tumor cells in circulation was unexpected. Using RP differential expression analysis, we compared CTCs with purified EGFP-tagged primary tumor single cells (TuGMP3) and bulk tumor samples (tumor cells admixed with reactive stromal cells). Six proteinaceous ECM genes were highly expressed by CTCs and by stromal component, but not by epithelial cells within primary tumors: *Dcn*, *Sparc*, *Ccdc80, Col1a2, Col3a1*, and *Timp2* (Figure 5A). RNA-ISH analysis of both *Dcn* and *Sparc* confirmed diffuse expression in stromal elements of mouse primary tumors, with rare areas where these transcripts are colocalized with keratin-expressing cells at the epithelial-stromal border (Figure 5B). *SPARC* is a well-known ECM protein gene found in stroma of human primary PDAC (Infante et al., 2007; Neuzillet et al., 2013; Sato et al., 2003). Indeed, RNA-ISH analysis of 198 primary human PDACs demonstrates abundant stromal cell expression of *SPARC* transcripts in 99% of cases, with up to a third of tumors with rare epithelial cells expressing this ECM gene product (Figure 5C). Consistent with these observations, RNA-seq of EGFP-tagged single primary tumor cells (Figure 5A) identified only 1 of 20 cells (5%) with coexpression of high levels (>100 rpm) of *Sparc* and *Krt19*. In summary, abundant expression of ECM genes is a common feature of all keratin-rich classical CTCs. This is in marked contrast to the primary tumor, where these gene products are secreted by supporting stromal cells and not by the epithelial cancer cells. However, rare cells at the epithelial-stromal interface of primary tumors do appear to express both keratins and ECM genes, consistent with the pattern observed in CTCs themselves.

### Human CTCs Express Diverse Proteinaceous ECM Genes

To confirm the expression of proteinaceous ECM genes by human cancer cells circulating in the bloodstream, we isolated single CTCs from patients with pancreatic (n = 7), breast (n = 29), and prostate (n = 77) cancers and subjected these to single-cell RNA-seq. Six ECM protein genes were highly expressed in human CTCs (>100 rpm in >15% of all CTC samples) (Figure 5D; Table S6). Notably, three genes (*SPARC*, *MGP*, *SPON2*) are ECM glycoproteins, defined as part of the core matrisome (Naba et al., 2012). The core matrisome protein *SPARC* was particularly enriched in pancreatic CTCs being expressed at high levels (>100 rpm) in 100% of pancreatic CTCs compared to 31% of breast and 9% of prostate CTCs. The notable differences in ECM protein gene expression across human epithelial CTCs suggest microenvironment tissue specificity as well as probable redundancies in ECM protein signaling. Together, the consistent expression of ECM gene family members in human CTCs suggests that their upregulation may contribute either to the generation of CTCs from primary tumors or to the survival of cancer cells deprived of microenvironmental signals as they circulate in the bloodstream.

### ECM Protein Gene SPARC Enhances Pancreatic Cancer Metastatic Potential

**Figure 6:**
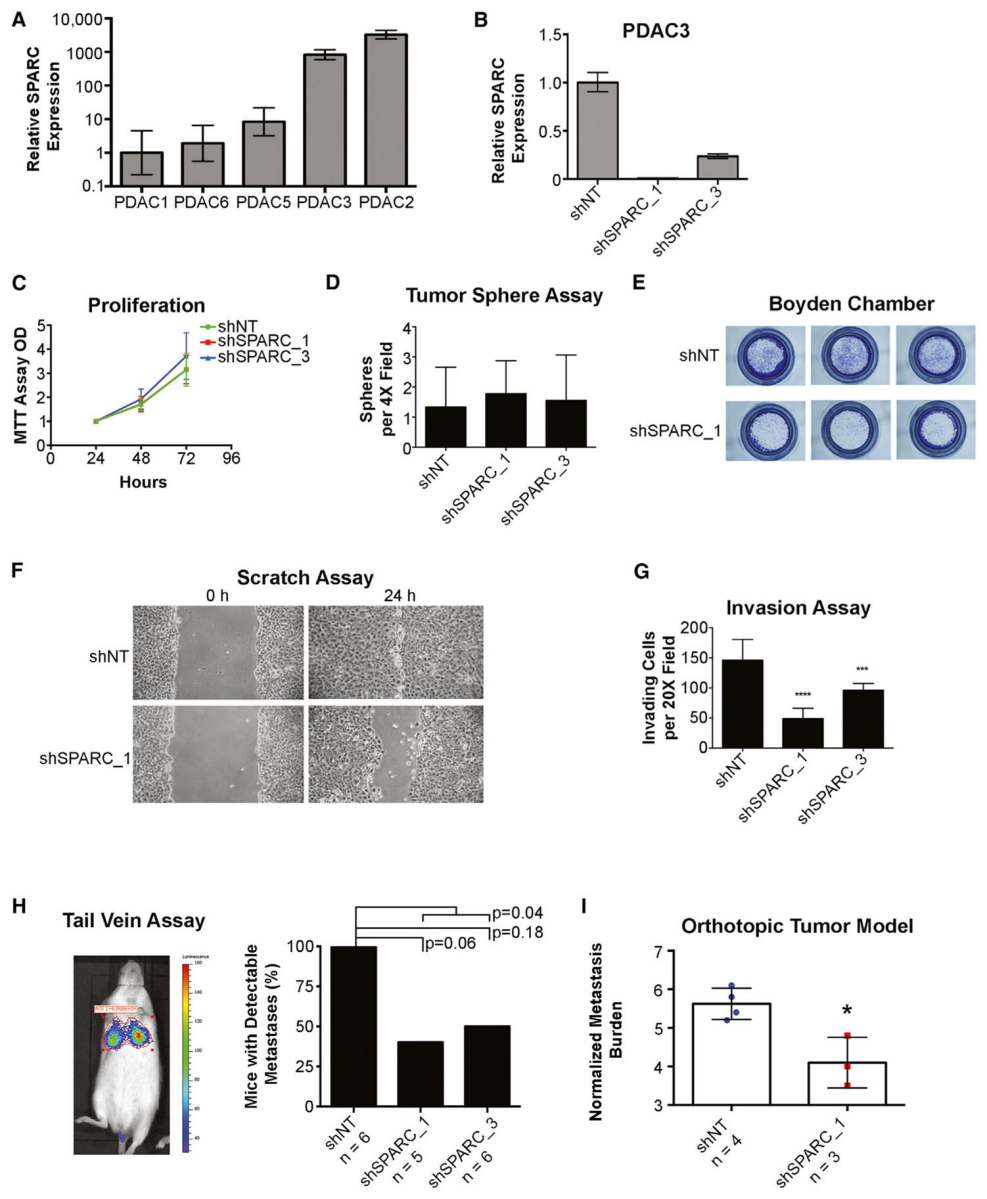
*SPARC* Expression in Human PDAC Enhances Invasion and Metastasis (A–C) Relative *SPARC* expression in (A) patient-derived human PDAC cell lines and in (B) PDAC3 cell line with shRNA against *SPARC* and nontarget (NT). Error bars represent range. (C) Proliferation of PDAC3 cell lines determined by MTT. (D) Tumor spheres in PDAC3 shNT versus shSPARC counted per 4× field (error bars represent SD). (E) Boyden migration chamber assay stained with crystal violet and imaged. (F) Scratch assay of shSPARC and shNT cell lines at 24 hr. (G) Invasion of shSPARC and shNT cell lines quantitated by number of nuclei/20× field. p value < 0.01 (**), 0.001 (***), 0.0001 (****). Error bars represent SD. (H) Percentage of detectable lung metastases by in vivo luciferase imaging after 3 weeks after tail vein inoculation of PDAC3 cell lines. Fisher’s exact test p value is shown. (I) Normalized metastasis burden in mice with orthotopic pancreatic tumors from PDAC3 cell lines. Error bars represent SD (*p < 0.05).

In order to define the functional consequences of *SPARC* expression in pancreatic cancer cells, we screened a panel of patient-derived, low-passage PDAC cell lines for expression. Two human PDAC cell lines with relatively high *SPARC* expression were identified (PDAC2 and PDAC3), making it possible to test the consequences of small hairpin RNA (shRNA)-mediated knockdown (Figures 6A, 6B, and S7). Suppression of endogenous *SPARC* expression in both PDAC2 and PDAC3 cell lines using two independent shRNA constructs did not affect proliferation in 2D cultures or anchorage-independent tumor sphere formation (Figures 6C, 6D, and S7). However, *SPARC* knockdown by both shRNAs significantly reduced pancreatic cancer cell migration in wound scratch assays and their invasive properties, as measured by in vitro Boyden assays (Figures 6E–6G and S7). Tail vein injection of *SPARC*-suppressed PDAC3 cells using both shRNA constructs generated significantly fewer lung metastases than cells expressing nontargeting hairpin (shNT) controls (Figure 6H). Metastases generated from orthotopic pancreatic xenografts were also significantly reduced for *SPARC*-suppressed PDAC3 cells, as measured by luciferase imaging and normalized for primary tumor size (Figure 6I). Thus, *SPARC* expression by pancreatic cancer cells appears to selectively enhance their invasive and migratory properties to augment metastatic virulence. The high levels of *SPARC* expression evident in virtually all pancreatic CTCs thus raises the possibility that it contributes significantly to the metastatic spread of pancreatic cancer.

## DISCUSSION

We present a detailed analysis of CTC composition and diversity in pancreatic cancer, using single-cell RNA-seq. We achieved high-quality transcriptomes in 93 single mouse pancreatic CTCs, which were compared with bulk and single-cell preparations from matched primary tumors and from an immortalized cell line established from the same mouse pancreatic tumor model. The use of the KPC mouse model made it possible to compare simultaneously isolated primary tumor specimens and CTCs, and it allowed measurements of CTC heterogeneity across multiple mice sharing the same *Kras*/*Trp53* genetic drivers. The large number of isolated CTCs and the high quality of the isolated RNA from these cells reflect the application of the CTC-iChip technology, which effectively depletes normal blood components, enriching for CTCs that are untagged and accessible for single-cell manipulation. Finally, the purification of CTCs irrespective of their cell-surface epitopes avoids any bias associated with their purification based on expression of common epithelial markers such as EpCAM.

**Figure 7:**
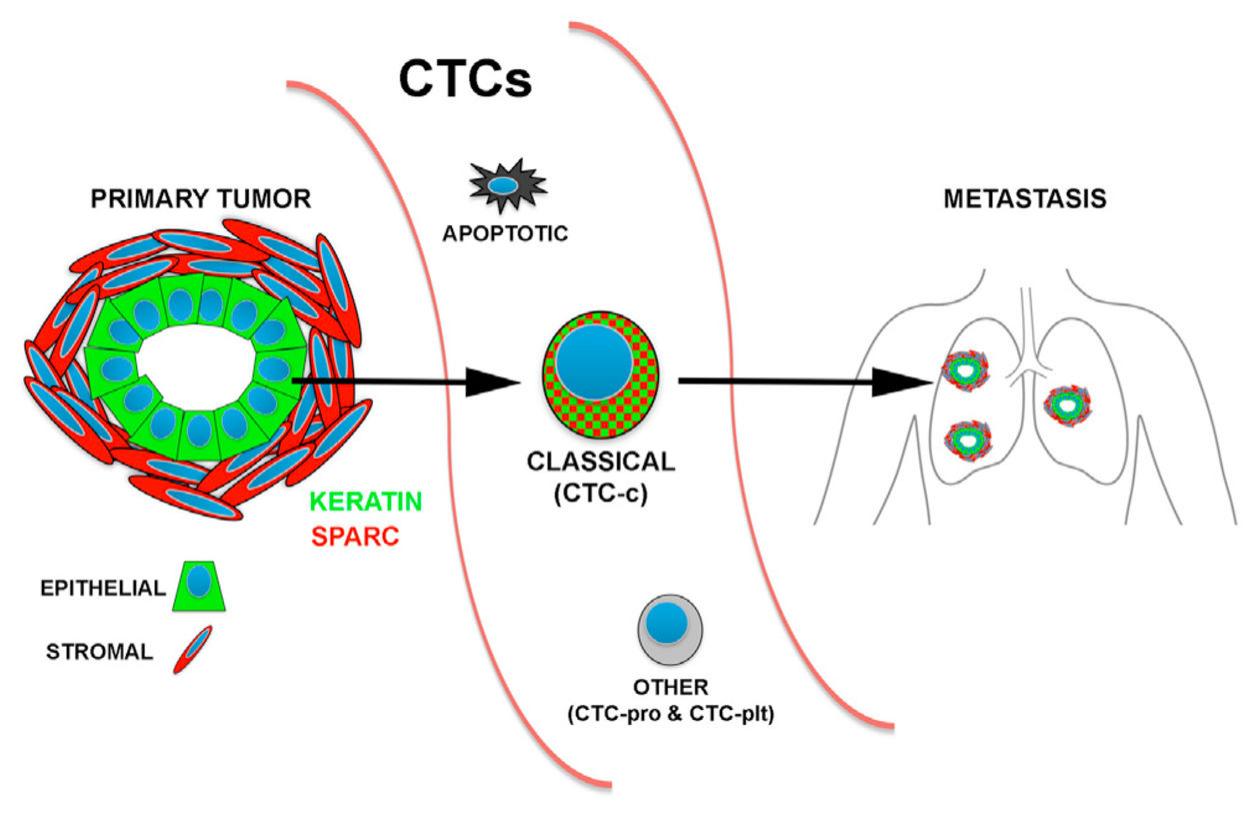
Summary Model of the Role of Pancreatic CTCs in the Metastatic Cascade Shown are the heterogeneous subsets of pancreatic CTCs with a focus on the most prominent classical CTC group, which are enriched for coexpression of epithelial (keratin) and stromal (*Sparc*) genes.

Together, our observations include the following. (1) CTC expression profiles cluster into three classes, including a major “classical CTC” group, and others that are defined by platelet-derived markers or proliferative signatures. (2) Common features shared by virtually all classical CTCs include expression of both epithelial and mesenchymal markers, the stem cell-associated gene *Aldh1a2*, and three highly expressed transcripts, *Klf4*, *Igfbp5*, and *Dcn*. The specific localization of *Igfbp5*-expressing cells at the epithelial-stromal boundary within primary tumors may point to a region that contributes significantly to CTC generation. (3) The most highly enriched CTC-specific transcripts shared by almost all classical CTCs encode extracellular matrix proteins, such as *Sparc*. (4) Aberrant expression in CTCs of this ECM gene product, which is normally abundant in the tumor stromal compartment, is observed in both mouse and human pancreatic CTCs, and its knockdown attenuates cancer cell migration and invasion in reconstituted systems. (Figure 7)

Compared with our previous RNA-seq of partially purified, bulk CTC populations, which required digital subtraction of leukocyte-derived reads (Yu et al., 2012, 2013), the single-cell analysis reported here provides considerably more depth of tumor cell-specific transcript reads, and it allows measurements of CTC heterogeneity. The feasibility of single-cell RNA-seq applied to CTCs has been reported for small numbers of immunoselected melanoma and prostate CTCs (Cann et al., 2012; Ramsköld et al., 2012), and our work extends these studies by providing a comprehensive landscape of mouse pancreatic CTCs, whose gene expression profile is directly compared to matched primary tumor cells. Since KPC mice primarily produce disseminated micrometastatic foci, we were unable to directly compare the expression profile of CTCs with that of metastatic lesions. The shared genetic drivers in the KPC mouse model enabled the collection and analysis of sufficient numbers of single CTCs across different animals, yet we note significant animal-specific clustering in RNA-seq data. Thus, in addition to the initiating mutations, somatically acquired genetic and epigenetic changes may distinguish CTCs derived from different tumors. Multiple mouse tumors contributed to each of the three distinct clusters of CTCs. Despite their atypical expression pattern, the identification of platelet-associated and proliferative CTC subsets as being tumor-derived is established by their inclusion of lineage-tagged tumor cells. The more characteristic expression pattern exhibited by the classical CTC cluster enabled detailed comparison with primary tumor cells, thereby providing further insight into the origin and properties of CTCs.

Mouse pancreatic classical CTCs uniformly lose expression of the epithelial marker E-cadherin (*Cdh1*), a key feature of epithelial-to-mesenchymal transition. However, the cells do not lose expression of other epithelial markers, such as cytokeratins, nor is there a consistent increase in classical mesenchymal markers such as vimentin. As such, most classical CTCs appear arrested in a biphenotypic state. Despite their expression of cytokeratins, which are present in the epithelial components of the primary tumor, most other highly expressed markers in CTCs are shared with the stromal component of the primary tumor. Among these stromal genes is *Aldh1a2*, a putative pancreatic cancer stem cell marker (Rasheed and Matsui, 2012; Rasheed et al., 2010). A provocative observation relating to the shared epithelial and mesenchymal state of classical CTCs is their virtually universal (93%) expression of *Igfbp5,* which is uniquely expressed in a small subpopulation of cells at the epithelial/stromal interface within primary tumors. This raises the possibility that this critical location within the primary tumor generates a disproportionate fraction of viable CTCs. The postulated role of human *IGFBP5* in metastasis (Hao et al., 2004) as well as in pancreatic malignancy (Johnson et al., 2006; Johnson and Haun, 2009) makes its unique expression pattern in both tumors and CTCs particularly noteworthy.

The most unexpected observation from our single-CTC RNA-seq study is the high abundance of ECM transcripts in the vast majority of classical CTCs. The coexpression of pancreatic cancer-enriched cytokeratins (*Krt7* and *Krt19*) in single cells expressing these ECM gene products excludes the possibility that these represent circulating tumor-derived fibroblasts. Interestingly, prior evaluation of matched primary and metastatic breast tumors identified the most prevalent gene expression difference as enrichment for ECM molecules in the metastases, comprising some 18% of differentially expressed genes (Weigelt et al., 2005). While this has been interpreted as reflecting differences in the local environment of the metastatic site, our data suggest that ECM proteins are highly expressed by CTCs themselves. By analogy with the classical “seed versus soil” debate (Fidler, 2003), CTCs may in fact be seeds carrying some of their own soil. These findings are also consistent with recent work highlighting the importance of tumor stromal signaling in priming cancer cells to metastasize (Zhang et al., 2013).

Consistent with the aberrant expression of *SPARC* in some pancreatic cancer cells, a subset of patient-derived tumor cell lines also coexpress it along with epithelial cytokeratins. The reduction in cell migration and metastatic potential exhibited by these pancreatic cell lines following *SPARC* knockdown suggests that it may contribute to CTC-mediated metastasis, consistent with prior work in breast cancer models (Minn et al., 2005). However, *Sparc* null pancreatic mouse tumors demonstrate some effects on collagen maturation but do not show suppressed metastasis (Neesse et al., 2014). Similar findings have been reported in prostate and breast *Sparc* null mouse cancer models (Wong et al., 2008). Thus, *Sparc* expression may contribute to metastasis, but inherent redundancies in ECM protein expression may mitigate this effect. Nonetheless, considerable effort has been directed to targeting the pancreatic cancer stroma as a means of improving delivery of chemotherapeutics as well as stripping tumor cells of their supportive microenvironment (Neesse et al., 2011; Olive et al., 2009; Provenzano et al., 2012; Rasheed et al., 2012). Our finding that these gene products are also expressed by CTCs themselves suggests a remarkable level of cellular plasticity. To the extent that invasive properties of CTCs are mediated in part by expression of such ECM proteins, it also raises the possibility of targeting cancer cells in the blood.

The ability to dissect critical components of the metastatic process at the single-cell level depends upon critical technological developments that have only recently become available, namely the efficient isolation of extraordinarily rare CTCs in solution without the bias of tumor antigen selection combined with the ability to perform high-fidelity single-cell RNA-seq. These approaches now allow CTC analyses to extend from matching them to known tumor-defining markers to interrogating them for unique properties that in fact distinguish them from primary tumor cells. Identifying such CTC-specific gene expression patterns may provide additional insight into mechanisms that underlie their ability to survive in the bloodstream and generate distant metastases, which are critical to the ultimate goal of preventing the spread of cancer to distant organs.

## EXPERIMENTAL PROCEDURES

### Mice and Cell Lines

Mice with pancreatic cancer used in these experiments express Cre driven by *Pdx1*, *LSL-Kras^G12D^*, and *Trp53^lox/+^* or *Trp53^lox/lox^* as previously described (Bardeesy et al., 2006). EGFP pancreatic lineage-tagged KPC mice were generated by breeding the mT/mG mouse [Jackson Laboratory; Gt(ROSA) 26Sortm4(ACTB-tdTomato,-EGFP)Luo/J] into the breeder pairs used for KPC mouse generation. Normal FVB mice were purchased from Jackson Laboratory. All mice care and procedures were done under Massachusetts General Hospital (MGH) Subcommittee on Research Animal Care-approved protocols.

### Human CTCs and Cell Lines

Human blood for CTC analysis was obtained after consent was obtained on an existing Dana-Farber/Harvard Cancer Center institutional review board (IRB)-approved protocol (05-300) at the Massachusetts General Hospital. A maximum of 20 ml of blood was obtained from patients at any given blood draw in two 10 ml EDTA tubes, and approximately 8–10 ml of blood was processed per patient. Newly derived pancreatic cancer cell lines were generated from metastatic ascites fluid from patients receiving diagnostic or therapeutic paracentesis under MGH IRB protocol 2011P001236. Cell lines were subcultured until a pure cell line was obtained. All cell lines studied had KRAS mutation genotyping to confirm cancer origins, and both PDAC2 and PDAC3 were both found to have KRAS G12V point mutations. Cell lines were grown in standard culture conditions using Dulbecco’s modified Eagle’s medium, high glucose + 10% fetal bovine serum + 1% penicillin/streptomycin (Gibco/Life Technologies).

### CTC Enrichment Technology

Given the desire for an unbiased enrichment system, the previously presented negative depletion technology was selected for this application. Before running blood, mouse and human blood were analyzed by a cell blood count machine to determine total WBC count. For mouse CTC samples, a rat anti-mouse CD45 antibody (BAM114, R&D Systems) was preconjugated to Dynabeads MyOne Strepavidin T1 (Life Technologies, 65602). Beads were added at a ratio of 125 beads/WBC, mixed, and incubated for 40 min at room temperature. Human CTC samples utilized a primary and secondary immunolabeling approach. Biotinylated primary antibodies against anti-human CD45 antibody (clone 2D1, R&D Systems, BAM1430) and anti-human CD66b antibody (Abd Serotec, 80H3) were spiked into whole blood at 100 fg/WBC and 37.5 fg/WBC, respectively, and incubated rocking at room temperature for 20 min. Dynabeads MyOne Strepavidin T1 (Life Technologies, 65602) were then added and incubated rocking at room temperature for an additional 20 min.

### Single-Cell Micromanipulation, Amplification, and Sequencing

After whole-blood CTC-iChip processing, the product containing enriched cells was collected in a 35 mm petri dish and viewed using a Nikon Eclipse Ti inverted microscope. Cells of interest were identified based on intact cellular morphology and lack of labeling with anti-CD45 magnetic beads. These target cells were individually micromanipulated with a 10 μm transfer tip on an Eppendorf TransferMan NK 2 micromanipulator and ejected into PCR tubes containing RNA protective lysis buffer and immediately flash frozen in liquid nitrogen. Single cells were amplified with a modified protocol (Tang et al., 2010) and sequenced on the ABI 5500XL system.

### RNA In Situ Hybridization

RNA-ISH was performed according to the Affymetrix ViewRNA ISH Tissue-2 Plex Assay. Fluorescent images were taken in using a Nikon 90i microscope. See Supplemental Experimental Procedures for details.

## Supplementary Material

Supplemental Figures and info

Supplemental Table 1

Supplemental Table 2

Supplemental Table 3

Supplemental Table 4

Supplemental Table 5

Supplemental Table 6

Supplemental table titles
