# Supplementary material for: Single-Cell RNA Sequencing Identifies Extracellular Matrix Gene Expression by Pancreatic Circulating Tumor Cells
Source: Cell Rep. Author manuscript; Available in PMC 2015 Sep 25. (PMC4230325; doi:10.1016/j.celrep.2014.08.029)
Supplement: Supplemental Figures and info [file NIHMS634041-supplement-Supplemental_Figures_and_info.pdf]

Figure S1

A

Mouse ID	Kras	Cre	Trp53	mT/mG	Gender	Age (Weeks)	Gross Metastatic Disease	IF CK+ CTC/mL Blood	CTCs Picked	CTCs Sequenced	Primary Single Cells Picked	Primary Single Cells Sequenced
MP1	G12D	Pdx1	L/L		M	5.86	No	118	3			
MP2	G12D	Pdx1	L/L		M	5.00	No	1694	36	13		
MP3	G12D	Pdx1	L/L		M	6.14	No	0	24	8		
MP4	G12D	Pdx1	L/L		F	8.00	Yes	28	42	16		
MP5	G12D	Pdx1	L/L		M	6.00	No	240	3			
MP6	G12D	Pdx1	L/L		F	6.43	No	861	24	16		
MP7	G12D	Pdx1	L/+		F	16.71	Yes	63	42	22		
GMP1	G12D	Pdx1	L/+	+/wt	M	8.86	No	N/A	12	7		
GMP2	G12D	Pdx1	L/+	+/+	M	10.00	No	N/A	12	11		
GMP3	G12D	Pdx1	L/+	+/wt	F	12.57	No	N/A	0		36	20
GMP4	G12D	Pdx1	L/+	+/wt	M	12.86	No	N/A	9	8		

B

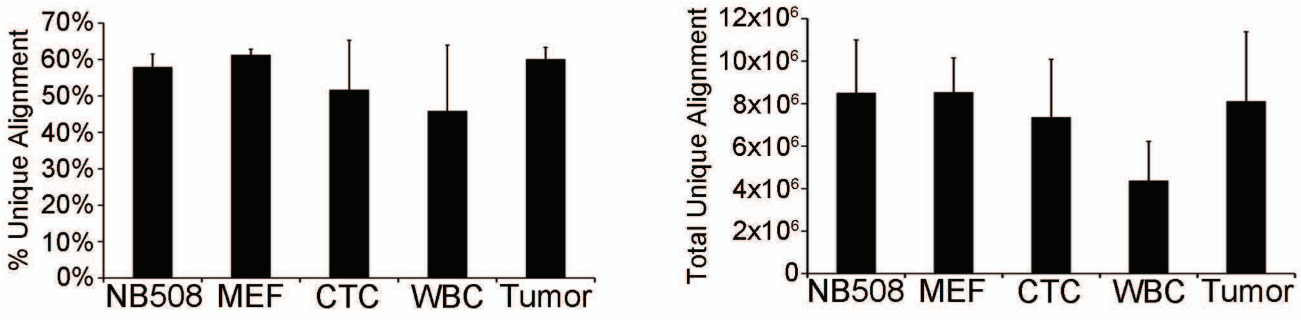

C

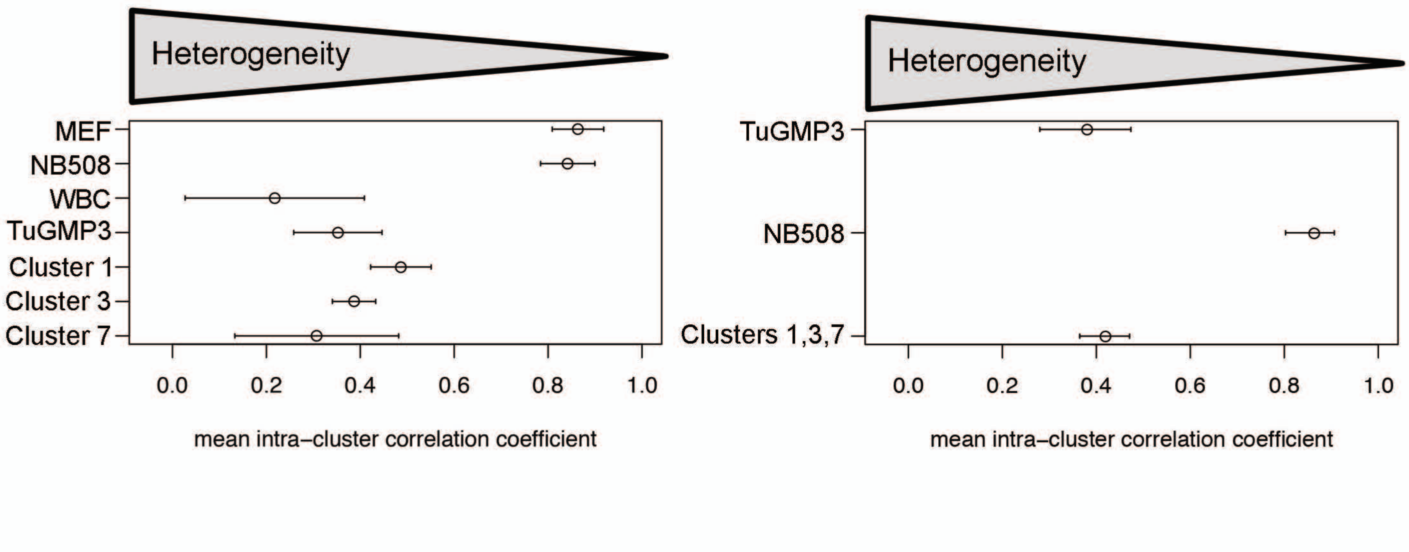

Figure S1, related to Figures 1 & 2: A) KPC mouse genotype and charcteristics. B) Quality metrics of single cell sequencing with % of reads aligned and total unique alignments for cell lines (NB508, MEF), CTCs, WBC, and diluted bulk RNA from matched primary tumors. C) Single cell heterogeneity using mean intra-cluster correlation coefficient for each cluster (left) and between single cell primary tumor (TuGMP3), cancer cell line (NB508), and all CTCs (Cluster 1,3,7) (right). Circle = mean, Range = 95% CI.

Figure S2

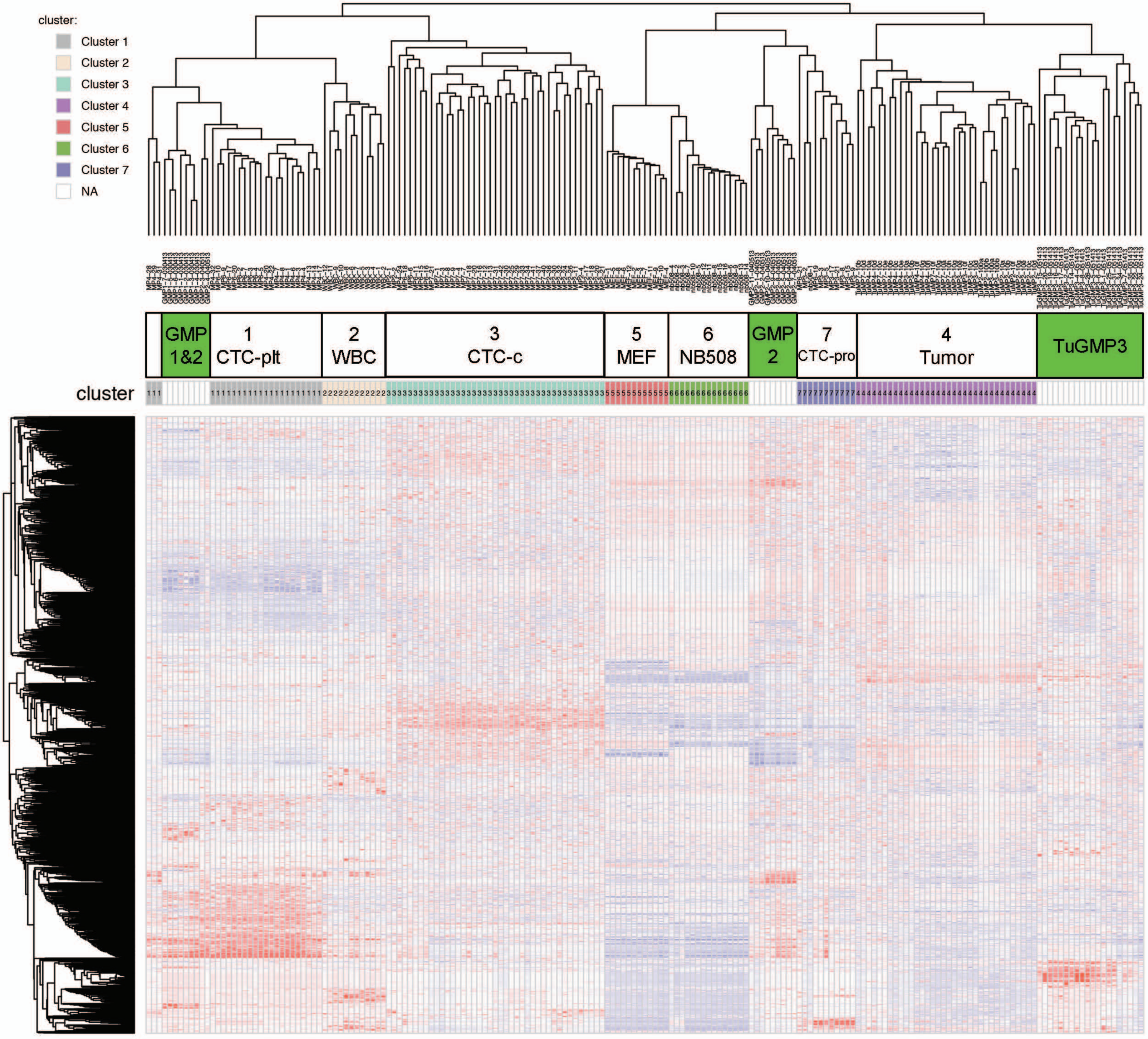

Figure S2, related to Figure 2: Unsupervised hierarchical clustering of all samples with inclusion of EGFP lineage labeled CTCs (GMP1 and GMP2) and single cell primary tumor cells (TuGMP3). GMP1 and GMP2 CTCs cluster within cluster 1 CTCs and other GMP2 cells cluster most closely with cluster 7 CTCs. TuGMP3 single primary tumor cells cluster closest with bulk tumor RNA from other KPC mice. Red is relative high and blue relative low expression.

Figure S3

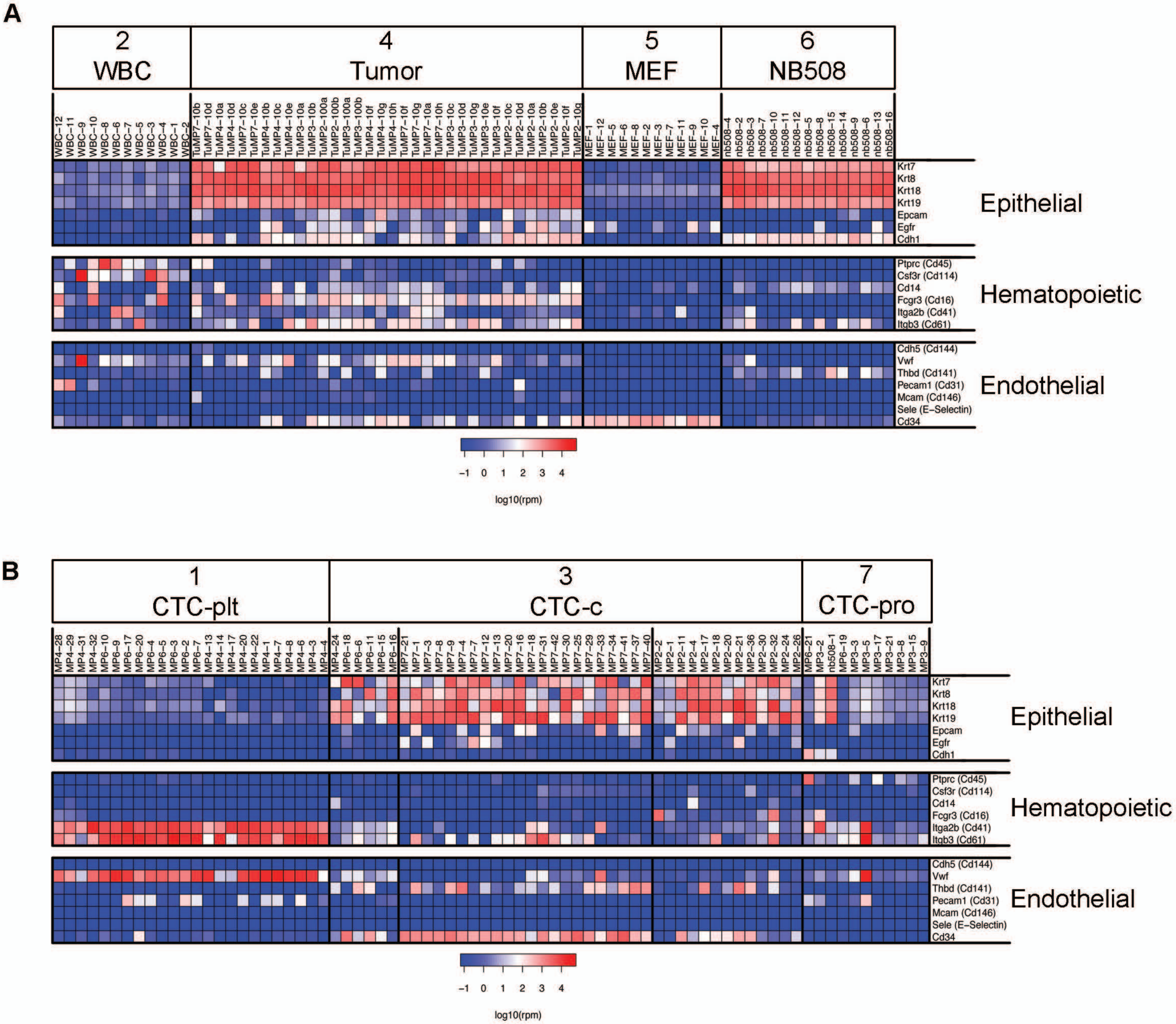

Figure S3, related to Figure 2: Expression heatmap of epithelial, hematopoietic, and endothelial markers in amplified sample A) controls (normal WBC, primary tumor, MEF, and NB508) and B) Candidate CTCs (Cluster 1, 3, 7). Scale in log10(rpm).

Figure S4

Remove Genes with |R| ≥ 0.6
(2838 genes)

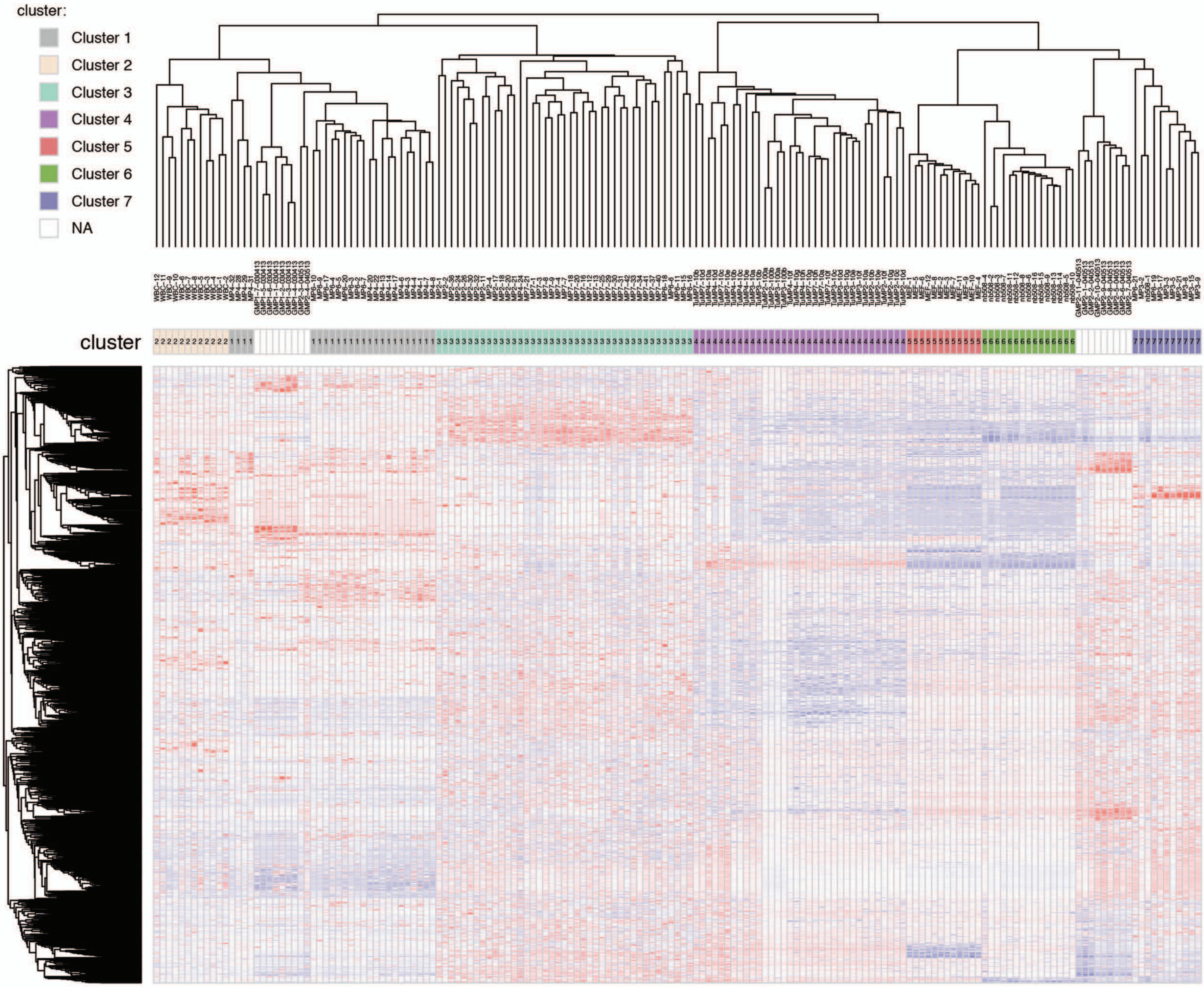

Figure S4, related to Figure 2: Digital Subtraction of Platelet Related Genes
Unsupervised hierarchical clustering of all samples after removal of cluster of genes enriched in platelet signatures primarily in Cluster 1 CTCs. Removal of these genes does not affect clustering of samples. Red is relative high and blue relative low expression.

Figure S5

A

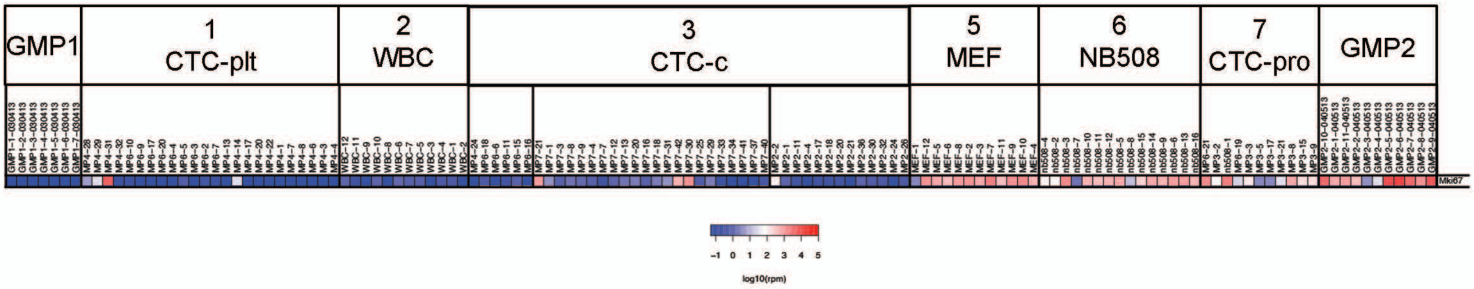

B

Whitefield Cell Cycle Literature

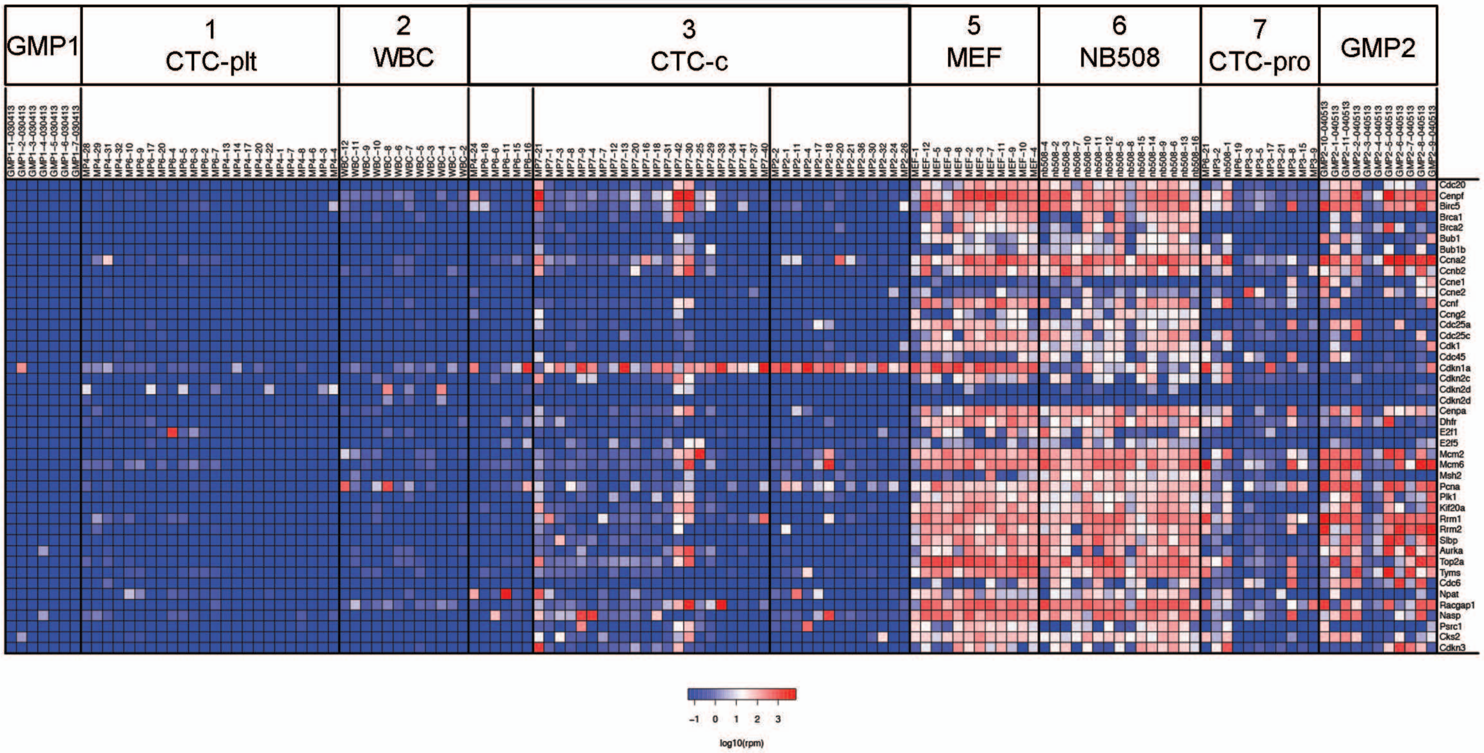

Figure S5, related to Figure 2: Proliferation markers elevated in cell lines and some CTCs. Heatmap of *Mki67* across single cell samples, which was enirched by RP in Cluster 7 (CTC-pro), MEF, NB508, and GMP2 relative to classical CTCs (CTC-c). Similar pattern is seen using MsigDB proliferation signature from Whitefield Cell Cycle Literature gene set. Scale in log10(rpm).

Figure S6

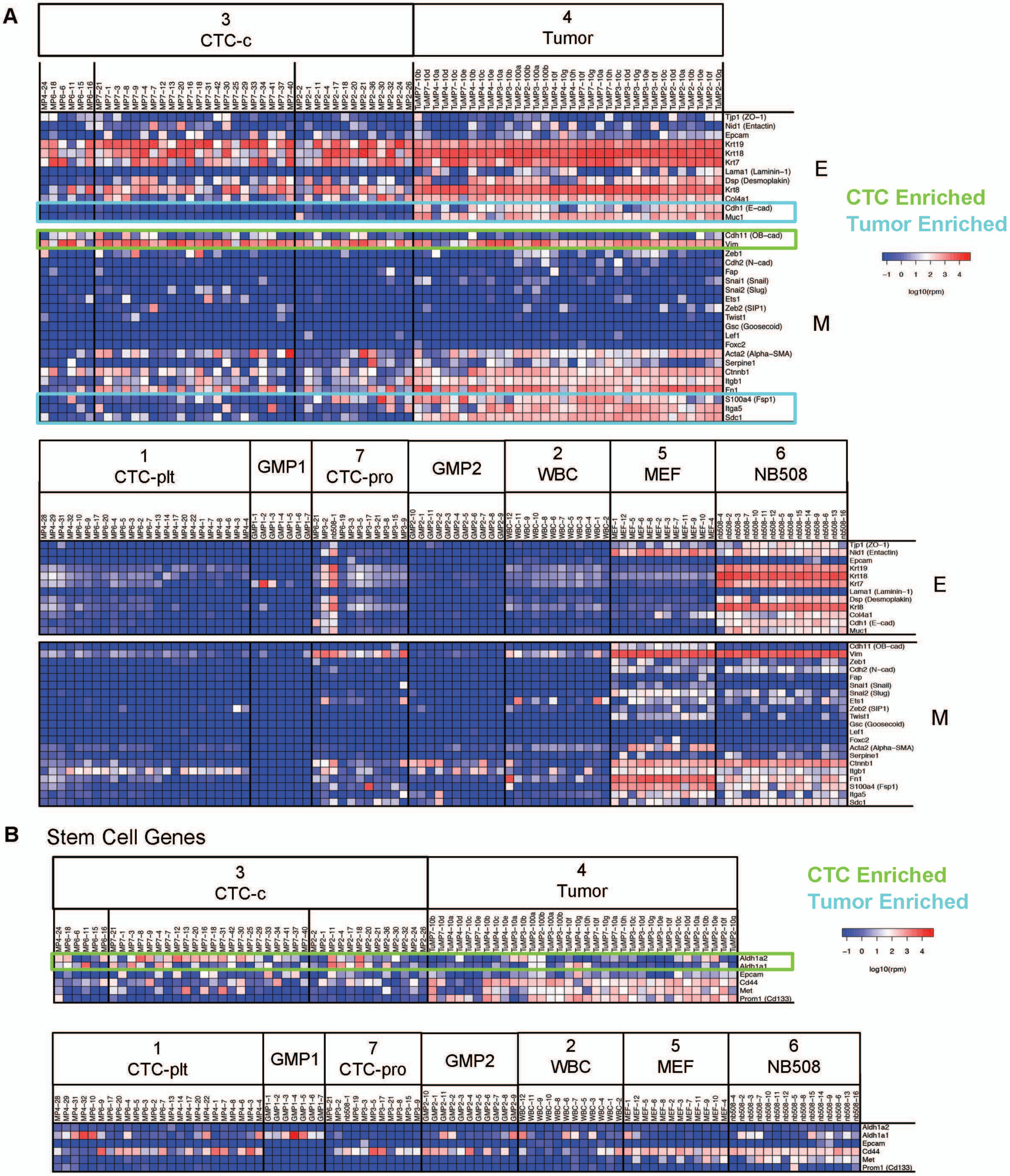

Figure S6, related to Figure 3: Expression of EMT and Stem Cell Genes in classical CTCs
Heatmap of A) EMT and B) Stem Cell genes for single cell data.
CTC-c Enriched (Light green box) and Tumor Enriched (Light blue box) genes by RP (FDR < 0.01)
Scale in log10(rpm).

Figure S7

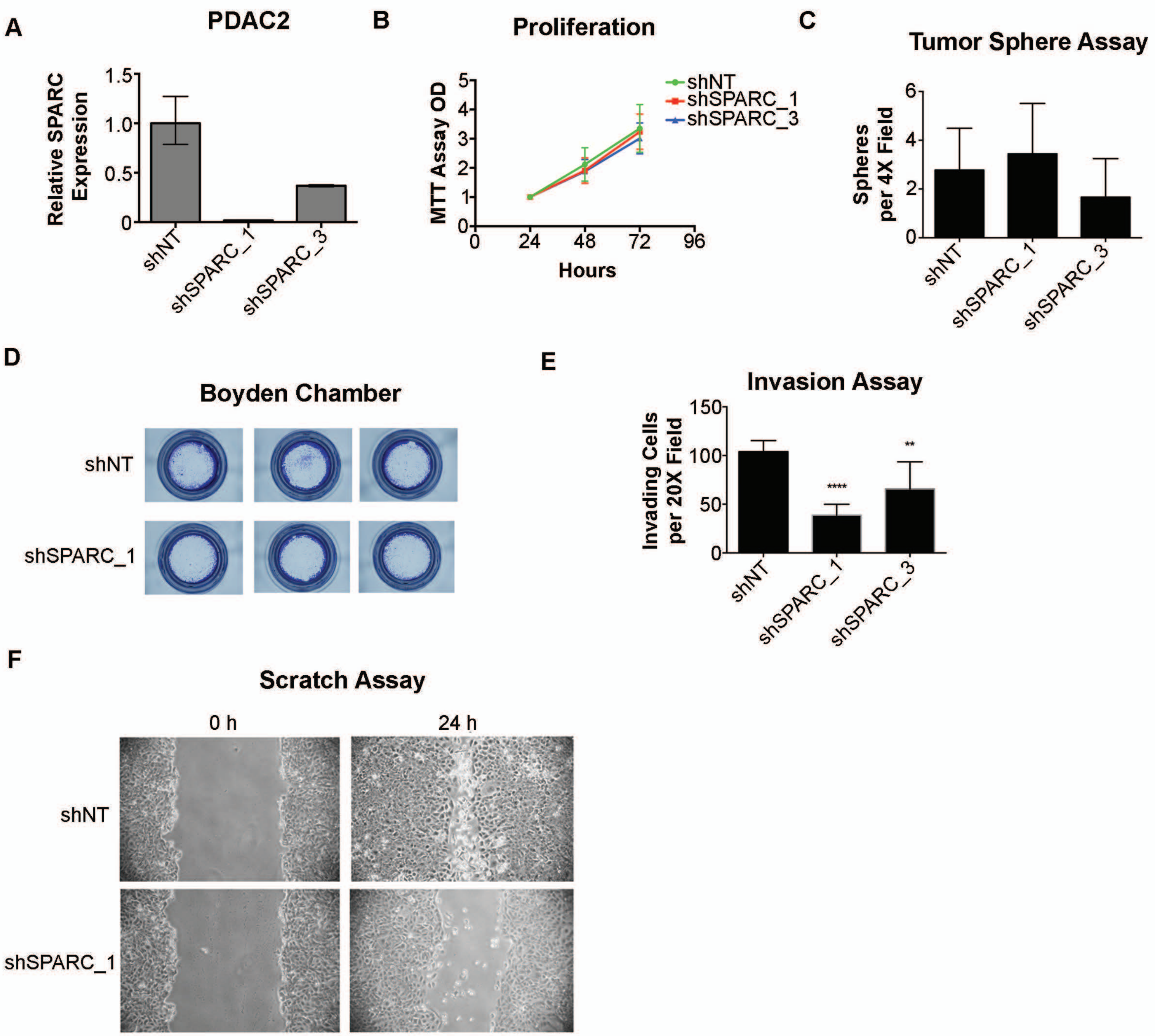

Figure S7, related to Figure 6: A) PDAC2 shRNA cell lines by qRT-PCR. Average shown with max and min RQ (error bars) B) Proliferation rates by MTT assay similar in PDAC2 cell line between shNT and shSPARC stable lines. C) Tumor sphere invasion assay (Error bars = std) formation at 2 weeks similar between shNT and shSPARC cell lines. Quantiation done per 4X magnification field. (Error bars = std). Migratory behavior reduced by shSPARC_1 & 3 as determined by D) Boyden chamber migration assay at 48 h, E) invasion assay at 48 h, and.by F) scratch wound healing assay at 24 h.

Supplemental Tables:

Table S1, related to Figures 2 & 3: Differentially expressed genes between groups by Rank Product

Table S2, related to Figures 2 & 3: Gene ontology analysis of CTC-c enriched genes compared to primary tumor

Table S3, related to Figures 2 & 3: KEGG analysis of CTC-c enriched genes compared to primary tumor

Table S4, related to Figure 2: Enriched gene sets for CTC-plt versus CTC-c

Table S5, related to Figure 2: Enriched gene sets for CTC-pro versus CTC-c

Table S6, related to Figure 5: Highly expressed ECM genes in human CTCs

Supplemental Experimental Procedures

Mice, tissue, cells, and cell lines

Mice with pancreatic cancer used in these experiments express Cre driven by *Pdx1*, *LSL-Kras*^{G12D}, and *Trp53*$^{lox/+}$ or *Trp53*$^{lox/lox}$ (otherwise referred to as KPC) as previously described (Bardeesy et al., 2006). EGFP pancreatic lineage tagged KPC mice were generated by breeding the mT/mG mouse (Purchased from the Jackson Laboratory - Gt(ROSA)26Sortm4(ACTB-tdTomato,-EGFP)Luo/J) into the breeder pairs used for KPC mouse generation. Normal FVB mice were purchased from Jackson Laboratory. All mice care and procedures were done under MGH SRAC approved protocols.

For cardiocentesis, animals were sedated with isofluorane, the chest wall was sterilized with ethanol and a skin incision was made above the rib cage to expose the thoracic cavity and eliminate normal skin epithelial cell contamination. A 23-gauge needle was used to draw approximately 1 mL of blood into a 1 mL syringe primed with 100 μL of PBS-10mM EDTA pH 7.4 (Gibco). Blood EDTA concentration was raised to 5mM by either the addition of a concentrated bolus of 500mM EDTA or 1:1 dilution with 10 mM EDTA. Animals were then euthanized per animal protocol guidelines.

A mouse pancreatic cell line NB508 (*Pdx1-Cre*/*Kras*^{G12D}/*Trp53*$^{lox/+}$) previously generated from primary tumors developed in this endogenous model was GFP transfected by lentivirus (NB508-GFP). This cell line was used for spiked cell experiments and orthotopic tumor formation.

NB508-GFP Cell lines were maintained in standard culture conditions using RPMI-1640 medium + 10% FBS + 1% Pen/Strep (Gibco/Life Technologies).

For orthotopic experiments, NB508-GFP cells were orthotopically injected into the pancreas of healthy syngeneic (FVB background) mice. Briefly, mice were anesthesized with isofluorane and the left abdominal wall was treated with Nair® hair removal product, and sterilized with 70% ethanol. A small incision was made on the upper left lateral abdominal wall and the pancreas was mobilized. Approximately 1 million NB508-GFP cells in PBS in a total volume of 0.1 mL was injected into the pancreas. The peritoneum and abdominal wall was closed by sterile surgical staples. The tumors were allowed to grow for 2 weeks, at which time blood was obtained by cardiocentesis for CTC-iChip processing.

Human blood for pancreatic CTC analysis was obtained after consent was obtained on an existing IRB protocol (05-300) at the Massachusetts General Hospital (MGH). A maximum of 20 mLs of blood was obtained from patients at any given blood draw in two 10 mL EDTA tubes.

Newly derived pancreatic cancer cell lines were generated from metastatic ascites fluid from patients receiving diagnostic or therapeutic paracentesis under MGH IRB protocol 2011P001236. Cell lines were sub-cultured until a pure cell line was obtained. All cell lines studied had KRAS mutation genotyping to confirm cancer origins and both PDAC2

and PDAC3 were both found to have KRAS G12V point mutations. Cell lines were grown in standard culture conditions using DMEM, high glucose + 10% FBS + 1% Pen/Strep (Gibco/Life Technologies).

CTC Enrichment Technology
Given the desire for an unbiased enrichment system, the previously presented negative depletion technology was selected for this application. Before running blood, mouse and human blood were analyzed by a cell blood count (CBC) machine to determine total WBC count.

For mouse samples, all processing protocols were identical to those previously identified, except a rat anti-mouse CD45 antibody (BAM114, R&D Systems, USA) was preconjugated to Dynabeads® MyOne^{TM} Strepavidin T1 (Life technologies 65602) for mouse CTC samples. Beads were added at a ratio of 125 beads/WB, mixed, and incubated for 40 minutes at room temperature.

Human samples utilized a primary and secondary immunolabeling approach. Biotinylated primary antibodies against anti-human CD45 antibody (Clone 2D1 R&D Systems BAM1430) and anti-human CD66b antibody (Abd Serotec 80H3) were spiked into whole blood at 100 fg/WBC and 37.5 fg/WBC respectively and incubated rocking at room temperature for 20 minutes. Dynabeads® MyOne^{TM} Strepavidin T1 (Life technologies 65602) were then added and incubated rocking at room temperature for an additional 20 minutes.

Spiked cell experiments were conducted to validate the system by spiking ~ 1000 GFP expressing NB508 cells into 1 mL of healthy mouse blood and processing to determine recovery efficiency. Orthotopic models were used to validate recovery efficiency as well as initially determine expected depletion efficiency from tumor-bearing mice. In these experiments, enriched samples were evaluated for the number of GFP+ cells observed in the product.

Immunostaining of CTCs Isolated from the endogenous model
Isolated CTCs were spun onto glass slides and immunostained using a primary-secondary approach. Primary antibodies were rabbit anti-wide spectrum cytokeratin (1:50, Abcam ab9377), and goat anti-mouse CD45 (1:500, R&D systems AF114). Secondary immunofluorescent-tagged antibodies were used for signal amplification. These were donkey anti-rabbit Alexa Fluor 594 (1:500, Invitrogen A-21207), and donkey anti-goat Alexa Fluor 488 (1:500, Invitrogen A-11055). Nuclei were then counterstained with DAPI and the slides were rinsed with PBS, cover slipped and stored at 4°C. They were imaged under 10x magnification using the BioView Ltd. automated imaging system (Billerica, MA) as well as an automated upright fluorescence microscope (Eclipse 90i, Nikon, Melville, NY). Positive staining for CK, without CD45 staining, was required for scoring potential CTCs, which were then manually reviewed. Threshold and baseline signals were established using specimens from non-tumor bearing mice.

Single cell micromanipulation
After whole blood anti-CD45 negative depletion, the product containing enriched cells was collected in a 35mm petri dish and viewed using a Nikon Eclipse Ti inverted fluorescent microscope. Cells of interest were identified based on intact cellular morphology and lack of labeling with anti-CD45 magnetic beads. These target cells were individually micromanipulated with a 10 µm transfer tip on an Eppendorf TransferMan® NK 2 micromanipulator and ejected into PCR tubes containing RNA protective lysis buffer (10X PCR Buffer II, 25mM MgCl2, 10% NP40, 0.1 M DTT, SUPERase-In, Rnase Inhibitor, 0.5 uM UP1 Primer, 10mM dNTP and Nuclease-free water) and immediately flash frozen in liquid nitrogen.

Human pancreatic CTCs were micromanipulated in a similar manner, but to enhance specificity a vital staining cocktail was used to select best single CTCs. CTC-iChip product was collected ina 1.5 mL Eppendorf tube on ice and spun at 2000 RPM for 3 min at 4° C in a microcentrifuge. The supernatant was aspirated and the pellet resuspended in 100 µL of chilled PBS 1%BSA with 2 µL of Alexa Fluor 488 conjugated anti-EpCAM (#5198 Cell Signaling Technology), 10 µl of Alexa Fluor 488 conjugated anti-CDH11 (#FAB17901G R&D Systems), and 5 µl each of Alexa Fluor 594 labeled anti-CD45 (#562279 BD Biosciences). After 15 minutes of incubation on ice, an additional 200 µL of chilled PBS 1%BSA was added and the product placed in a 35 mm culture dish for single cell micromanipulation based on AlexaFluor 488 positive (Green) and AlexaFluor 594 negative (Red) criteria.

Single Cell Amplification and Sequencing
Single cell amplification and sequencing were done as previously described (Tang et al., 2010) with slight modifications underlined below. RNA samples from extracted single circulating tumor cells were thawed on ice and incubated at 70°C for 90 seconds. To generate cDNA, samples were treated with reverse transcription master mix (0.05 uL RNase inhibitor, 0.07uL T4 gene 32 protein, and 0.33uL SuperScript III Reverse Transcriptase per 1X volume) and incubated on thermocycler at 50°C for 30 minutes and 70°C for 15 minutes. To remove free primer, 1.0uL of EXOSAP mix was added to each sample, which was incubated at 37°C for 30 minutes and inactivated at 80°C for 25 minutes. Next, a 3'-poly-A tail was added to the cDNA in each sample by incubating in master mix (0.6uL 10X PCR Buffer II, 0.36uL 25mM $MgCl_2$, 0.18uL 100mM dATP, 0.3uL Terminal Transferase, 0.3uL RNase H, and 4.26uL H_2O per 1X volume) at 37°C for 15 minutes and inactivated at 70°C for 10 minutes. A second strand cDNA was synthesis by dividing each sample into 4 and incubating in master mix (2.2uL 10X High Fidelity PCR Buffer, 1.76uL 2.5mM each dNTP, 0.066uL UP2 Primer at 100uM, 0.88uL 50mM $MgSO_4$, 0.44uL Platinum Taq DNA Polymerase, and 13.654uL H_2O per 1X volume) at 95°C for 3 minutes, 50°C for 2 minutes, and 72°C for 10 minutes.

PCR amplification (95°C for 3 minutes, 20 cycles of 95°C for 30 seconds, 67°C for 1 minute, and 72°C for 6 minutes 6 seconds) was performed with master mix (4.1uL 10X High Fidelity PCR Buffer, 1.64uL 50mM $MgSO_4$, 4.1uL 2.5mM each dNTP, 0.82uL AUP1 Primer at 100uM, 0.82uL AUP2 Primer at 100uM, 0.82uL Platinum Taq DNA Polymerase, and 6.7uL H_2O per 1X volume). The 4 reactions of each sample were pooled

and purified using the QIAGEN PCR Purification Kit (Cat. No 28106) and eluted in 50uL EB buffer. Samples were selected by testing for genes Gapdh, ActB, Ptprc (CD45), Krt8, Krt18, Krt19, and Pdx1 using qPCR. Each sample was again divided in 4 and a second round of PCR amplification (9 cycles of 98°C for 3 minutes, 67°C for 1 minute, and 72°C for 6 minutes 6 seconds) was performed with master mix (9uL 10X High Fidelity PCR Buffer, 3.6uL 50mM $MgSO_4$, 13.5uL 2.5mM each dNTP, 0.9uL AUP1 Primer at 100uM, 0.9uL AUP2 Primer at 100uM, 1.8uL Platinum Taq DNA Polymerase, and 59.1uL H_2O per 1X volume). Samples were pooled and purified using Agencourt AMPure XP beads and eluted in 40uL 1X low TE buffer.

Sequencing Library Construction
To shear the DNA using the Covaris S2 System, 1X low TE buffer and 1.2uL shear buffer were added to each sample. Conditions of the shearing program include: 6 cycles, 5°C bath temperature, 15°C bath temperature limit, 10% duty cycle, intensity of 5, 100 cycles/burst, and 60 seconds. Then, samples were end-polished at room temperature for 30 minutes with master mix (40uL 5X Reaction Buffer, 8uL 10mM dNTP, 8uL End Polish Enzyme1, 10uL End Polish Enzyme2, and 14uL H_2O per 1X volume). DNA fragments larger than 500bp were removed with 0.5X volumes of Agencourt AMPure XP beads. Supernatant was transferred to separate tubes. To size-select 200-500bp DNA products, 0.3X volumes of beads were added and samples were washed 2X with 70% EtOH. The products were eluted in 36uL low TE buffer. A dA-tail was added to each size-selected DNA by treating with master mix (10uL 5X Reaction Buffer, 1uL 10mM dATP, and 5uL A-Tailing Enzyme I per 1X volume) and incubated at 68°C for 30 minutes and cooled to room temperature. To label and distinguish each DNA sample for sequencing, barcode adaptors (5500 SOLiD 4464405) were ligated to DNA using the 5500 SOLiD Fragment Library Enzyme Module (4464413). Following barcoding, samples were purified twice using the Agencourt AMPure XP beads and eluted in 22uL low TE buffer. Following a round of PCR Amplification (95°C for 5 minutes, 12 cycles of 95°C for 15 seconds, 62°C for 15 seconds, and 70°C for 1 minute, and 70°C for 5 minutes), the libraries were purified with AMPure XP beads. Finally, to quantify the amount of ligated DNA, SOLiD Library TaqMan Quantitation Kit was used to perform qPCR. Completed barcoded libraries were then subjected to emulsion PCR with template beads preparation and sequenced on the ABI 5500XL.

Determination of reads-per-million (rpm)
Color space reads were aligned using tophat version 2.0.4 (Trapnell et al., 2009) and bowtie1 version 0.12.7 with the no-novel-juncs argument set with mouse genome version mm9 and transcriptome defined by the mm9 knownGene table from genome.ucsc.edu. Reads that did not align or aligned to multiple locations in the genome were discarded. The mm9 table knownToLocusLink from genome.ucsc.edu was used to map, if possible, each aligned read to the gene whose exons the read had aligned to. The reads count for each gene was the number of reads that were so mapped to that gene. This count was divided by the total number of reads that were mapped to any gene and multiplied by one million to form the reads-per-million (rpm) count. We used rpm rather than rpkm because this sequencing protocol utilizes an oligo-dT reverse transcription method whereby there is only one cDNA per transcript causing a 3' bias in the alignments.

Expression heatmap generation

Genes selected for visualization as an expression heatmap were done using Grid Graphics in R. To characterize candidate CTCs we utilized established epithelial markers commonly used in the CTC field (*Krt7, Krt8, Krt18, Krt19, Epcam, Egfr, Cdh1*), established hematopoietic markers (*Ptprc/Cd45, Csf3r/Cd114, Cd14, Fcgr3/Cd16, Itga2b/Cd41, Itgb3/Cd61*) utilizing the BD biosciences CD marker handbook (http://www.bdbiosciences.com/documents/cd_marker_handbook.pdf), and endothelial markers (*Cdh5/Cd144, Vwf, Thbd/Cd141, Pecam1/Cd31, Mcam/Cd146, Sele/E-selectin, Cd34*) based on literature (Bertolini et al., 2006; Strijbos et al., 2008).

For hematopoietic markers, initial analysis was done using the common leukocyte marker (*Ptprc/Cd45*) and specific leukocyte subset markers for T-cells (*Cd3d, Cd3e, Cd3g, Cd247, Cd4, Cd8a, Cd8b1*), B-cells (*Cd19, Cd20*), granulocytes (*Cd15, Csf3r/Cd114*), monocytes (*Csf3r/Cd114, Cd14, Fcgr3/Cd16*), NK cells (*Fcgr3/Cd16, Ncam1/Cd56*), and platelets (*Itga2b/Cd41, Itgb3/Cd61*). Hematopoietic markers chosen for publication were based on evaluating these markers and showing representative markers with expression in normal WBCs sequenced as well as ones with high expression in any CTC sample (i.e. CTC-Plt expression of *Itga2b/Cd41* and *Itgb3/Cd61*).

Epithelial mesenchymal transition genes as well as pancreatic stem cell genes were selected from two reviews (Kalluri and Weinberg, 2009; Rasheed and Matsui, 2012).

Unsupervised hierarchical clustering and principal components analysis

The minimum of 1 and the smallest positive value of the rpm matrix was added to the rpm matrix to eliminate zeros. The result was then log10 transformed, yielding what we will call the log10(rpm) matrix. The rows (corresponding to genes) of the log10(rpm) matrix with the top 2000 standard deviations were retained and the rest of the rows discarded. The result was then median polished. The result was clustered using agglomerative hierarchical clustering with average linkage with distance metric equal to 1 minus the Pearson correlation coefficient. The principal components of the log10(rpm) matrix were computed and the coordinates of the samples with respect to the first three principal components were plotted.

Measures of cellular heterogeneity

For a collection of clusters of samples, we defined a statistic, M, as the mean over the clusters of the mean over all the pairs of samples in the cluster of the atanh of the correlation coefficient between the two columns of the rpm matrix corresponding to the pair. The "mean intra-cluster correlation coefficient" was defined as $\tanh(M)$. We used the jackknife estimator with respect to the samples to estimate a standard deviation, s, of the statistic. The 95% CI was defined as $\tanh(M \pm s\Phi^{-1}(0.975))$, where Φ is the cumulative distribution function of the standard normal distribution. To compute a p-value for the null hypothesis that the mean of the distribution of the M statistic for a cluster is the same as the mean of the distribution of the M statistic for a collection of clusters, we let $p = 2\left(1 - \Phi\left(|M_1 - M_2|/\sqrt{s_1^2 + s_2^2}\right)\right)$. Of note, we performed bootstrap

on the same data as an alternative to jackknife and we obtained similar results (data not shown).

Supervised differential gene expression using rank product

To find differentially expressed genes between two sets of samples, we started with the log10(rpm) matrix defined above. We removed columns corresponding to samples not in either set of samples. We then removed rows for which the 90th percentile of the values was less than log10(10). We then used the RP function of the Bioconductor (Gentleman et al., 2004) RankProd package (version 2.28.0) to get FDR estimates for both up and down differential expression. We considered genes to be differentially expressed if their FDR estimate was less than 0.01, but discarded genes that were both up and down differentially expressed, if there were any.

Gene set enrichment

We considered enrichment in four gene set collections: (1) all of KEGG, as found in DAVID 6.7 (Huang da et al., 2009), (2) Gene Ontology (GO) using GO_BP as found in DAVID 6.7,and (3) GO_CC as found in DAVID 6.7. Sets of genes found to be differentially expressed were tested for enrichment in the gene set collections using a hypergeometric test for each gene set in the collection. The resulting p-values for each collection were converted to FDR estimates using the Benjamini-Hochberg method (Benjamini and Hochberg, 1995).

Expression boxplots

p-values for all expression boxplots were computed using a two-sided two-sample Wilcoxon test. In boxplots that compared three classes, the p-values for the three possible comparisons were corrected for multiple-hypothesis testing using the method of Holm (Holm, 1979).

Digital removal of all annotated platelet transcripts

We removed from the log10(rpm) matrix (defined above) the 446 genes whose expression in the log10(rpm) matrix had an absolute value of correlation coefficient greater than 0.6 with the expression of any of the genes in the gene sets named GNATENKO_PLATELET_SIGNATURE and TENEDINI_MEGAKARYOCYTE_MARKERS in MSigDB v3.1.
We then clustered as described above.

RNA in situ Hybridization (RNA-ISH)

Paraffin-embedded tissue blocks were freshly cut and frozen at -80°C. Upon removal from the freezer, slides were baked for 1 hr at 60°C and fixed in %10 formaldehyde for 1 hr at room temperature (RT). Paraffin was removed using Histo-Clear and RNA-ISH was performed according to the Affymetrix ViewRNA ISH Tissue-2 Plex Assay. Tissue sections were permeabilized by pretreating in buffer solution for 10 min at 95°C and digestedwith protease for 10 min, before being fixed at RT in 5% formaldehyde. Target probe sets were applied and hybridized to the tissue by incubating for 2 hr at 40°C. For mouse samples, type 1 probes were used at a dilution of 1:50 and included Aldh1a2 (VB1-14197), Dcn (VB1-14962), Klf4 (VB1-14988), Igfbp5 (VB1-14987), and Sparc

(VB1-14196). Type 6 probes included EGFP (VF6-13336) at 1:50 and pooled Krt8 (VB6-11060) and Krt18 (VB6-11059) at 1:100 each. For human samples, type 1 SPARC (VA1-11122) was used at 1:50 with Type 6 probes KRT7, 8, 18, 19 (VA6-11562, VA6-11560, VA6-11561, VA6-10947) pooled each at 1:200. Signal was amplified through the sequential hybridization of PreAmplifier and Amplifer QT mixes to the target probe set. Target mRNA molecules were detected by applying Type 6 Label Probe with Fast Blue substrate and Type 1 Label Probe with Fast Red substrate. Tissue was counterstained with Gill's Hematoxylin for 10 sec at RT. DAPI (Invitrogen, D3571; 3.0 μg/ml) staining was performed for 1 min. Fluorescence microscopy using a Nikon 90i was used to visualize target mRNAs. Type 1 probes were detected in the Cy3 channel and Type 6 probes in the Cy5 channel. Merged images were generated using NIS-Elements software.

Quantitative RT-PCR
Total RNA (2 μg) was reverse transcribed using a cDNA synthesis kit (Superscript III, Invitrogen). Quantitative RT-PCR was performed using standard methods using validated Taqman human SPARC and GAPDH probes.

Constructs and Viral Infection
To produce replication incompetent lentivirus, 293T cells were co-transfected with either non-targeting pLKO.1 (shNT) or SPARC shRNA constructs in pLKO.1 (shSPARC), in combination with VSVG and Δ168 (Addgene) using Lipofectomine 2000 reagent (Invitrogen). 24 hours later, cell media was changed. Viral supernatants were harvested at 48 hours post-transfection and concentrated with LentiX solution. Viral pellets were resuspended in 400 μL base media. PDAC lines were infected overnight with 50 μL lentivirus in 8 μg/mL polybrene. Puromycin (3 μg/mL) was used to select transduced cells. Selection was complete 7 days after infection. Knockdown of SPARC expression was measured by qRT-PCR. Human shSPARC and shNT were obtained from the MGH Molecular Profiling Laboratory and originally from the Broad/MIT lentiviral RNAi library (Moffat et al., 2006).

Sequences for shRNA are as follows
shSPARC-1: 3'UTR 5'- CGGTTGTTCTTTCCTCACATT-3'
shSPARC_3: CDS 5'- CCAGGTGGAAGTAGGAGAATT -3'

GFP/Luciferase lentivirus was produced by co-transfecting the third generation lentivirus vector with REV, VSVG and PDML using Lipofectamine 2000 reagent. Virus was produced as described above. Infected cells were sorted on the basis of GFP expression using FACS.

Proliferation Assay
Cells (3000) were plated in a 96-wells. Every 24 hours for 7 days, 50 uL of 5 mg/mL Thiazolyl Blue Tetrazoluim Bromide (MTT) reagent was added to each well, incubated for 2 hours at 37°C and absorbance was measured at 595 nm.

Scratch Assay

Plate cells in a confluent monolayer. 24 hours after plating, scratch cells with a p200 pipette tip to create a straight line devoid of cells. Wash cells once with PBS. Add fresh media and image. Monitor and image every 24 hours for wound closure.

Transwell Migration and Invasion Assays
Cells ($1x10^4$) were plated on 8 μM pore transwell chambers with or without growth factor-reduced Matrigel (BD Biosciences). After 48 hours, invasion assays were terminated. Non-invading cells were removed from the top of the transwells by washing and swabbing. Invasive and/or migrating cells were quantified by fixing chambers in 4% paraformaldehyde for ten minutes and staining with Crystal violet (migration) nuclear DAPI (invasion). For each transwell, three 10X fields were imaged and counted in triplicate wells.

Tumor Sphere Assay
Cells were plated as single cell suspension in ultralow attachment 6-well (Corning) and grown in RPMI medium (serum free) supplemented with 1X B27 (Invitrogen), 20 ng/ml EGF and 20 ng/ml bFGF. Fresh media (1 ml or 30 μl) was added every 7 days. Tumor spheres were counted and photographed at day 14.

Tail Vein Metastasis Assay
The animal protocol was approved by the MGH Subcommittee on Research Animal Care. Six-week old NOD *scid* gamma (NSG) mice were anesthetized by isofluorane, 5×10^5 PDAC3 cells expressing shNT, shSPARC_1, or shSPARC3 in 100 μl of PBS were into the tail vein. Metastatic tumor formation in the lung was monitored weekly by bioluminescence using IVIS Lumina II (Caliper Life Science) after injection of 150 μL RediJect D-Luciferin (Perkin Elmer #760505).

Xenograft Metastasis Assay
To confirm the role of *SPARC* in metastasis, we created pancreatic orthotopic tumors by injecting $1x10^6$ GFP-luciferase tagged PDAC-3 cells with or without stable knockdown of the *SPARC* gene (PDAC-3 shSPARC_1 vs PDAC-3 shNT) in 12 immunodeficient mice (NSG mice). Tumor burden was quantified every 7-days by bioluminescent IVIS imaging as described above. Five weeks following the orthotopic injection, the mice were sacrificed to assess metastatic burden.

Normalized metastatic tumor burden was calculated by normalizing the metastatic tumor burden of each mouse by its relative tumor burden. Relative light units (RLU) were obtained from the primary tumor, liver, lung, and any visceral metastases found in the animal. The following equations were used to determine the normalized metastatic tumor burden.

Total Metastatic Burden for Each Mouse$_{ni}$=Liver_RLU + Lung_RLU + Visceral_RLU

RLU_Primary Tumor$_{n1}$ = Mouse with the lowest primary tumor burden

Relative Primary Tumor Burden for Each Mouse (1; 2; …, *ni*) => RLU_Primary Tumor burden$_{\mathbf{n1}}$/RLU_Primary Tumor burden$_{\mathbf{n1}}$; RLU_Primary Tumor$_{\mathbf{n2}}$/RLU_Primary Tumor burden$_{\mathbf{n1}}$; RLU_Primary Tumor burden$_{\boldsymbol{ni}}$/RLU_Primary Tumor burden$_{\mathbf{n1}}$

Normalized Total Metastatic Burden for Each Mouse (1; 2; …, *ni*) = Total Metastatic Burden Mouse$_{\boldsymbol{ni}}$ / Relative Primary Tumor Burden

Log10 transformation of the data was performed and unpaired t-test was used to assess the statistical significance.

Three mice were excluded from the analysis. Two mice (one from the shNT group and one from the shSPARC_1 group) were excluded due to extra-pancreatic tumor implantation (peritoneal implantation). A third mouse was excluded due to poor luciferin absorption resulting in unevaluable tumor burden.

Supplemental Methods References

Bardeesy, N., Aguirre, A.J., Chu, G.C., Cheng, K.H., Lopez, L.V., Hezel, A.F., Feng, B., Brennan, C., Weissleder, R., Mahmood, U*., et al.* (2006). Both p16(Ink4a) and the p19(Arf)-p53 pathway constrain progression of pancreatic adenocarcinoma in the mouse. Proc Natl Acad Sci U S A *103*, 5947-5952.

Benjamini, Y., and Hochberg, Y. (1995). Controlling the False Discovery Rate: A Practical and Powerful Approach to Multiple Testing. Journal of the Royal Statistical Society Series B (Methodological) *57*, 289-300.

Bertolini, F., Shaked, Y., Mancuso, P., and Kerbel, R.S. (2006). The multifaceted circulating endothelial cell in cancer: towards marker and target identification. Nat Rev Cancer *6*, 835-845.

Gentleman, R.C., Carey, V.J., Bates, D.M., Bolstad, B., Dettling, M., Dudoit, S., Ellis, B., Gautier, L., Ge, Y., Gentry, J*., et al.* (2004). Bioconductor: open software development for computational biology and bioinformatics. Genome biology *5*, R80.

Holm, S. (1979). A Simple Sequentially Rejective Multiple Test Procedure. Scandinavian Journal of Statistics *6*, 65-70.

Huang da, W., Sherman, B.T., and Lempicki, R.A. (2009). Systematic and integrative analysis of large gene lists using DAVID bioinformatics resources. Nat Protoc *4*, 44-57.

Kalluri, R., and Weinberg, R.A. (2009). The basics of epithelial-mesenchymal transition. J Clin Invest *119*, 1420-1428.

Moffat, J., Grueneberg, D.A., Yang, X., Kim, S.Y., Kloepfer, A.M., Hinkle, G., Piqani, B., Eisenhaure, T.M., Luo, B., Grenier, J.K*., et al.* (2006). A lentiviral RNAi library for human and mouse genes applied to an arrayed viral high-content screen. Cell *124*, 1283-1298.

Rasheed, Z.A., and Matsui, W. (2012). Biological and clinical relevance of stem cells in pancreatic adenocarcinoma. Journal of gastroenterology and hepatology *27 Suppl 2*, 15-18.

Strijbos, M.H., Gratama, J.W., Kraan, J., Lamers, C.H., den Bakker, M.A., and Sleijfer, S. (2008). Circulating endothelial cells in oncology: pitfalls and promises. Br J Cancer *98*, 1731-1735.

Tang, F., Barbacioru, C., Nordman, E., Li, B., Xu, N., Bashkirov, V.I., Lao, K., and Surani, M.A. (2010). RNA-Seq analysis to capture the transcriptome landscape of a single cell. Nat Protoc *5*, 516-535.

Trapnell, C., Pachter, L., and Salzberg, S.L. (2009). TopHat: discovering splice junctions with RNA-Seq. Bioinformatics *25*, 1105-1111.
