# Supplementary material for: Single-Cell RNA Sequencing Identifies Extracellular Matrix Gene Expression by Pancreatic Circulating Tumor Cells
Source: Cell Rep. Author manuscript; Available in PMC 2015 Sep 25. (PMC4230325; doi:10.1016/j.celrep.2014.08.029)
Supplement: Supplemental table titles [file NIHMS634041-supplement-Supplemental_table_titles.pdf]

**Supplemental Tables:**

Table S1, related to Figures 2 & 3: Differentially expressed genes between groups by Rank Product

Table S2, related to Figures 2 & 3: Gene ontology analysis of CTC-c enriched genes compared to primary tumor

Table S3, related to Figures 2 & 3: KEGG analysis of CTC-c enriched genes compared to primary tumor

Table S4, related to Figure 2: Enriched gene sets for CTC-plt versus CTC-c

Table S5, related to Figure 2: Enriched gene sets for CTC-pro versus CTC-c

Table S6, related to Figure 5: Highly expressed ECM genes in human CTCs
